# A randomised trial of malaria vaccine R21/Matrix-M™ with and without antimalarial drugs in Thai adults

**DOI:** 10.1038/s41541-024-00920-1

**Published:** 2024-07-06

**Authors:** Borimas Hanboonkunupakarn, Mavuto Mukaka, Podjanee Jittamala, Kittiyod Poovorawan, Pongphaya Pongsuwan, Lisa Stockdale, Samuel Provstgaard-Morys, Kesinee Chotivanich, Joel Tarning, Richard M. Hoglund, Natenapa Chimjinda, Katie Ewer, Fernando Ramos-Lopez, Nicholas P. J. Day, Arjen M. Dondorp, Adrian V. Hill, Nicholas J. White, Lorenz von Seidlein, Sasithon Pukrittayakamee

**Affiliations:** 1 Mahidol Oxford Tropical Medicine Research Unit, Faculty of Tropical Medicine, Mahidol University, Bangkok, Thailand; 2 Department of Clinical Tropical Medicine, Faculty of Tropical Medicine, Mahidol University, Bangkok, Thailand; 3 Nuffield Department of Medicine, Centre for Tropical Medicine and Global Health, University of Oxford, Oxford, UK; 4 Department of Tropical Hygiene, Faculty of Tropical Medicine, Mahidol University, Bangkok, Thailand; 5 The Jenner Institute Laboratories, University of Oxford, Oxford, UK; 6 GSK, GSK Vaccines Institute for Global Health, Siena, Italy; 7 Centre for Clinical Vaccinology and Tropical Medicine, The Jenner Institute, University of Oxford, Oxford, UK; 8 Present Address: The Royal Society of Thailand, Dusit, Bangkok, Thailand

**Keywords:** Translational research, Outcomes research

## Abstract

In preparation for mass vaccinations with R21/Matrix-M™ combined with mass administrations of dihydroartemisinin, piperaquine, and a single low dose primaquine we assessed the tolerability, safety, and potential interactions of this combination affecting immunogenicity or pharmacokinetics. 120 healthy Thai volunteers were randomised to receive either antimalarials combined with vaccinations (*n* = 50), vaccinations alone (*n* = 50), or antimalarials only (*n* = 20). Three rounds of vaccines and antimalarials were administered one month apart. The vaccine was well tolerated alone and in combination with the antimalarials. None of the participants failed completion of the 3-dose vaccine course. There was no significant difference in the vaccine immunogenicity or in the pharmacokinetics of piperaquine given individually or in combination. This study supports proceeding to a large trial of mass vaccinations with R21/Matrix-M™ combined with mass antimalarial administration in Bangladesh.

## Introduction

By the end of 2023 two pre-erythrocytic malaria vaccines have been prequalified by the WHO for purchase by UN organisations and other funders. Both vaccines induce immune responses against the circumsporozoite protein (CSP) a dominant antigen of sporozoites, the first stage of *P. falciparum* after entering the human host after a mosquito bite. Both vaccines confer pre-erythrocytic stage protection but have no blood stage or transmission blocking activity. Both vaccines include virus-like particles in which Hepatitis B surface antigen (HBsAg) is fused to CSP albeit in different proportions.

RTS,S/AS01 (Mosquirix, GlaxoSmithKline), the first malaria vaccine that was licensed and WHO prequalified, confers moderate levels of protection against falciparum malaria for a limited time. Over 18 months of follow-up, a three-dose regimen of RTS,S/AS01 conferred 46% protection against clinical malaria in 5 to 17 month old children, and 27% in 6 to 12 week old infants^1^. During months 38 to 48 of follow-up vaccine efficacy decreased to 28% and 18% respectively^2^. The protection was improved by combining vaccinations with antimalarial drug administrations^3^. By combining a four-dose regimen of RTS,S/AS01 with seasonal malaria chemoprevention (SMC; monthly treatment doses of amodiaquine-sulphadoxine-pyrimethamine) administered just before the malaria season, the protective efficacy of the vaccine -SMC combination as compared with chemoprevention alone was 63% against clinical malaria, 71% against hospital admission with severe malaria, and 73% against death from malaria in trial sites in Mali and neighbouring Burkina Faso^4^.

R21/Matrix M is the second licensed and WHO prequalified malaria vaccine. It is biosimilar to RTS,S/AS01 but with a higher CSP to HbsAg ratio. In a phase 3 trial in four African countries R21/Matrix-M™ was found to be well tolerated and safe. The vaccine efficacy over a 12 months period was 75% in sites with seasonal malaria where antimalarial chemoprevention is deployed and 67% in sites with perennial falciparum malaria transmission^5,6^. The production capacity for RTS.S/AS01 is limited and insufficient for global implementation. In contrast, the technology for R21/Matrix-M™ has been transferred to the Serum Institute of India Private Ltd (SIIPL, Pune, India), which has the capacity to produce large quantities of the vaccine at low cost.

In sub-Saharan Africa where the malaria burden is greatest in young children, malaria vaccination is being integrated into national childhood vaccinations programmes. This vaccination strategy is not appropriate in Asia where falciparum malaria transmission is low, highly heterogeneous, and presents mainly in adult populations with occupational exposure to malaria vectors. Importantly, malaria-endemic hotspots in Asia are the breeding ground for anti-malarial drug resistance. Malaria vaccination could be employed as an additional and essential tool in malaria elimination in such areas. We propose to evaluate the combined strategy of mass drug administrations with mass R21/Matrix-M™ vaccination campaigns. Before such a strategy can be implemented, the performance of R21/Matrix-M™ with antimalarial drugs needs to be assessed. We aimed to: (1) assess the tolerability, safety, and immunogenicity of R21/Matrix-M™ in Thai adults, (2) confirm that the co-administration of antimalarial drugs with the malaria vaccine R21/Matrix-M™ does not reduce the immunogenicity of the vaccine, and (3) assess the pharmacokinetics of the antimalarial drug piperaquine given in this study in combination with dihydroartemisinin and a single low dose of primaquine (SLDPQ) in subjects who concurrently receive R21/Matrix-M™.

## Results

**Fig. 1:**
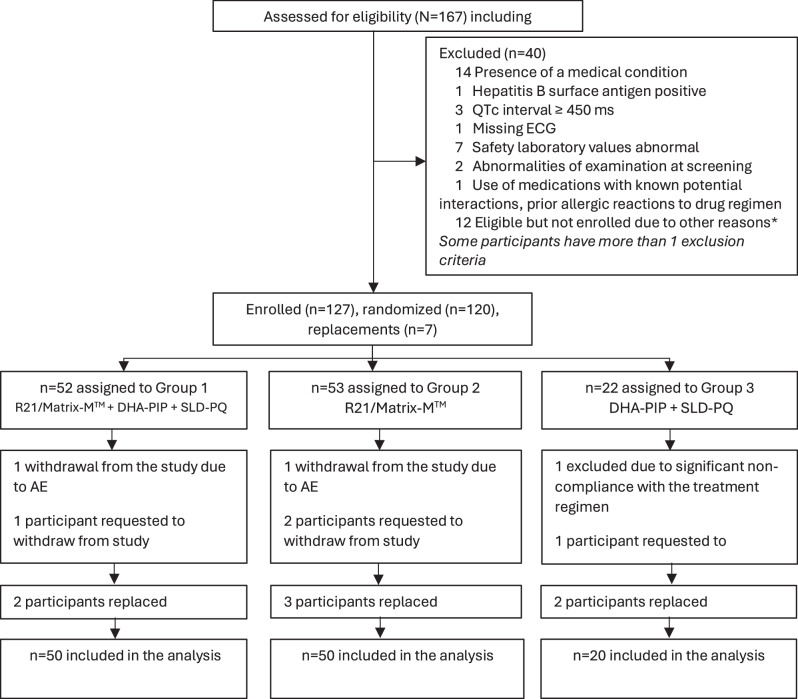
Trial profile. The Trial profile illustrates the assembly of study participants.

167 participants were screened between 4th January 2023 and 31st March 2023. Of these, 127 participants were enroled including seven participants who withdrew before the end of their follow-up and were replaced as per the study design. Two of the 7 withdrawn participants were in Group 1; 3 in Group 2 and 2 in Group 3 (Fig. 1). The main reason for exclusion was the presence of a medical condition (14/167; 8%).

### Baseline characteristics

A total of 120 participants were recruited and had complete follow-up (Table 1). The median age was 40 years (IQR 32 to 44 years). 39 of 120 study participants (33%) were male. The median weight of the study participants was 63.1 kg (IQR 54.1 to 71.7 kg). The mean and standard deviation (SD) of haemoglobin were 13.1 g/dL and 2.6 g/dL respectively. The mean temperature and SD were 36.4 °C and 0.3 °C respectively. None of the participants had fever at baseline.

**Table 1:** Characteristics (M0 Day 0 measurements) of the participants by vaccine group

Characteristics	Group 1 *n* = 50	Group 2 *n* = 50	Group 3 *n* = 20	Total *n* = 120
Gender *n* (% female)	31 (62)	35 (70)	15 (75)	81 (67.5)
Age (years): median (IQR)	40.5 (32, 45)	40 (32, 44)	40.5 (31.5, 44)	40 (32, 44)
Weight (kg): median (IQR)	64.3 (56, 71.3)	60.7 (52.1, 73)	64 (53.3, 71.7)	63.1 (54.1, 71.7)
QTc int (msec): median (IQR)	416 (405, 426)	415.5 (401, 430)	416 (402, 429)	416 (403.5, 428.5)
Fever *n* (%)	0	0	0	0
Hb (g/dL): mean (SD)	13.5 (3.9)	12.9 (1.2)	12.7 (1.1)	13.1 (2.6)
WBC (10^3^/µL): median (IQR)	6.4 (5.7, 7.6)	7.1 (6, 7.9)	6.7 (5.3, 7.9)	6.5 (5.8, 7.6)
Platelets (10^3^/µL): median (IQR)	281.5 (241, 307)	271 (238, 333)	262.5 (241, 310)	277 (240.5, 325.5)
Creatine (mg/dL): median (IQR)	0.7 (0.7, 0.9)	0.7 (0.7, 0.8)	0.7 (0.6, 0.8)	0.7 (0.7, 0.9)
AST (U/L): median (IQR)	20.5 (17, 26)	17.5 (16, 20)	19.5 (16.5, 21.5)	19 (16, 22)
ALT (U/L): median (IQR)	19 (14, 26)	15.5 (12, 21)	14 (12.5, 19)	17 (13, 23.5)
Temperature (°C): mean (SD)	36.4 (0.4)	36.4 (0.3)	36.5 (0.4)	36.4 (0.3)

Group 1 = R21/Matrix-M™ + DHA-PIP + SLD-PQ *n* = 50, Group 2 = R21/Matrix-M™ *n* = 50, Group 3 = No vaccine *n* = 20.

*IQR* interquartile range, *sd* standard deviation, *QTc int* corrected QT interval, *Hb* haemoglobin, *WBC* White Blood cell Count, *AST* Aspartate aminotransferase, *ALT* alanine transaminase.

### Safety analysis

Of 120 participants 82 had at least one solicited (including general and local) or unsolicited AE (Table 3 and Tables S1–3). Of these, 38 were in Group 1, 39 in Group 2, and 5 in Group 3. The proportions of solicited local and general adverse events (AEs) within seven days were similar between Group 1 (74% (95% CI 59.7–85.4)) and Group 2 (72% (95% CI 57.5–83.8)) as shown in Table 2. There were no solicited AEs in the no vaccine group (Group 3) as shown in Tables 2 and 3. The proportions of unsolicited AEs from 1st vaccination to Day 28 after the last vaccine dose was higher in Group 1 compared to Group 2 (Fisher’s exact *p* = 0.016). There was no significant difference in the proportion of unsolicited AEs from 1st vaccination to 28 days after last vaccine between Group 1 and Group 3 (Fisher’s exact *p* = 0.387) and between Group 2 and Group 3 (Fisher’s exact *p* = 0.456; Table 2). Most of the AEs were mild to moderate in severity. Two participants developed severe general solicited AEs, namely headache and muscle pain. All recovered within two days. Seven participants had severe unsolicited AEs two of which were SAEs. Six study participants had recovered by the end of the study. One participant developed anaemia, which was considered not related to vaccine and/or antimalarials. One SAE occurred in Group 1 (2%, 95% CI 0.1–10.6). This participant was a 46-year-old male who was hospitalised with community-acquired pneumonia 12 days after the first dose of vaccine and antimalarials. *H. influenzae* and *K. pneumoniae* were detected in the sputum and the participant recovered with antibiotics. This pneumonia episode was considered not related to vaccine. The second SAE was in Group 3 (5%; 95% CI 0.1–24.9). The participant was a 46-year-old female who was hospitalised with neuromyelitis optica. This episode was a relapse. The participant had failed to divulge during screening previous episodes of optic neuritis.

**Table 2:** Number of participants with at least 1 solicited local or general AEs within 7 days of vaccination (cumulative over month 0, month 1, and month 2)

Safety parameter	Group 1 *n* = 50	Group 2 *n* = 50	Group 3 *n* = 20	Total *n* = 120
Solicited local and general AEs within seven days: *n*(%), (95% CI)	37 (74), (59.7, 85.4)	36 (72), (57.5, 83.8)	0	73 (60.8), (51.5, 69.6)
Unsolicited AEs: date of 1^st^ vaccine to 28 days after last vaccine: *n*(%), (95% CI)	17 (34.0), (21.2, 48.8)	6 (12.0), (4.5, 24.3)	4 (20.0), (5.7, 43.7)	27 (22.5), (15.4, 31)
SAEs within 28 days after each vaccine: *n*(%), (95% CI)	1 (2.0), (0.1, 10.6)	0	1 (5.0), (0.1, 24.9)	2 (1.7), (0.2, 5.9)
SAEs for whole period: *n*(%), (95% CI)	1 (2.0), (0.1, 10.6)	0	1 (5.0), (0.1, 24.9)	2 (1.7), (0.2, 5.9)
AEs or SAEs leading to withdrawal from further vaccination from Dose 1 to study conclusion: *n*(%), (95% CI)	0	0	0	0
pIMDs from Dose 1 to study conclusion: *n*(%), (95% CI)	0	0	1 (5.0), (0.1, 24.9)	1 (0.8), (0.0, 4.6)
Meningitis from Dose 1 to study conclusion: *n*(%), (95% CI)	0	0	0	0

Group 1 = R21/Matrix-M™ + DHA-PIP + SLD-PQ *n* = 50, Group 2 = R21/Matrix-M™ *n* = 50, Group 3 = No vaccine *n* = 20.

*AE* adverse event, *SAE* serious adverse event, *pIMD* potential immune mediated disorder.

**Table 3:** Overall general adverse events by vaccine group: Number of participants with at least 1 solicited general AE within 7 days of the vaccine over month 0, month 1 and month 2

General symptoms	Group 1 *n* = 50	Group 2 *n* = 50	Group 3 *n* = 20	Total *n* = 120
Muscle pain: *n*(%), (95% CI)	18 (36), (22.9, 50.8)	23 (46), (31.8, 60.7)	0	41 (34.2), (25.8, 43.4)
Fatigue: *n*(%), (95% CI)	21 (42), (28.2, 56.8)	17 (34), (21.2, 48.8)	0	38 (31.7), (23.5, 40.8)
Headache: *n*(%), (95% CI)	18 (36), (22.9, 50.8)	15 (30), (17.9, 44.6)	0	33 (27.5), (19.7, 36.4)
Fever: *n*(%), (95% CI)	12 (24), (13.1, 38.2)	7 (14), (5.8, 26.7)	0	19 (15.8), (9.8, 23.6)
Nausea: *n*(%), (95% CI)	13 (26), (14.6, 40.3)	4 (8), (2.2, 19.2)	0	17 (14.2), (8.5, 21.7)
Chills: *n*(%), (95% CI)	10 (20), (10.0, 33.7)	6 (12), (4.5, 24.3)	0	16 (13.3), (7.8, 20.7)
Diarrhoea: *n*(%), (95% CI)	11 (22), (11.5, 35.9)	3 (6), (1.3, 16.5)	0	14 (11.7), (6.5, 18.8)
Joint pain: *n*(%), (95% CI)	7 (14), (5.8, 26.7)	7 (14), (5.8, 26.7)	0	14 (11.7), (6.5, 18.8)
Vomiting: *n*(%), (95% CI)	4 (8), (2.2, 19.2)	1 (2), (0.1, 10.6)	0	5 (4.2), (1.4, 9.5)

Group 1 = R21/Matrix-M™ + DHA-PIP + SLD-PQ *n* = 50, Group 2 = R21/Matrix-M™ *n* = 50, Group 3 = No vaccine *n* = 20.

### Piperaquine plasma concentrations

**Fig. 2:**
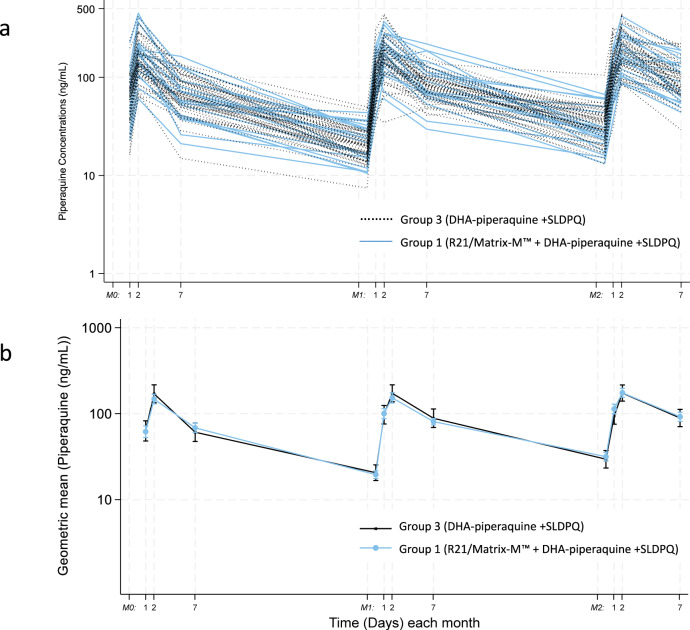
Piperaquine concentrations in adult Thai study participants receiving dihydroartemisinin-piperaquine with or with the malaria vaccine R21/Matrix-M™. **a** The line chart shows the Individual trajectories of piperaquine concentrations. **b** The line chart summarises the geometric mean with 95% confidence intervals of the trajectories of piperaquine concentrations.

We performed individual trajectory plots of piperaquine plasma concentrations at each day of the month when the concentrations were measured. As shown in Fig. 2 below, there is a good overlap in trajectories between the individuals in Group 1 and Group 3.

There were no significant differences in the distribution of the piperaquine plasma concentrations between Group 1 and Group 3 (Table 4).

**Table 4:** Distribution of piperaquine concentrations by month and day of measurement (median; IQR: Q1 to Q3)

Month	Day	Group 3 *n* = 20 (ng/mL)	Group 1 *n* = 50 (ng/mL)	*P* value (Wilcoxon rank sum test)
0	0	Below limit of quantification	Below limit of quantification	NA
0	1	67.0 (43.9–89.1)	64.9 (44.5–88.7)	0.900
0	2	177.5 (125.0–213.5)	139.0 (114.0–185.0)	0.201
0	7	57.9 (40.8–86.5)	68.2 (53.0–91.7)	0.361
0	AUC 0-7d ng*h/mL	30,594.4 (21,015.2–36,429.7)	23,151.6 (19,577.8–33,561.8)	0.198
1	0	22.4 (15.0–29.9)	19.8 (15.3–24.6)	0.572
1	1	99.0 (59.0–162.5)	104.0 (74.6–139.0)	0.814
1	2	177.0 (134.5–246.0)	153.5 (113.0–202.0)	0.302
1	7	82.2 (66.8–5 128.5)	82.1 (64.2–98.0)	0.508
1	AUC 0-7d ng*h/mL	30,672.1 (20,366.4–38,646.6)	24,221.4 (16,812.2–31,600.8)	0.121
2	0	27.4 (21.8–46.8)	33.8 (24.7–40.1)	0.460
2	1	92.6 (66.6–131.5)	111.5 (83.5–152.0)	0.155
2	2	169.5 (116.0–257.0)	179.0 (136.0–231.0)	0.967
2	7	80.5 (56.7–134.5)	86.5 (68.8–114.0)	0.635
2	AUC 0-7d ng*h/mL	28,055.4 (19,286.0–42,462.6)	27,652.0 (21,772.0 to 38,006.9)	0.723

Group 1 = R21/Matrix-M™ + DHA-PIP + SLD-PQ *n* = 50; Group 3 = No vaccine *n* = 20.

*NA* not applicable, *AUC* area under curve.

### Immunogenicity

**Fig. 3:**
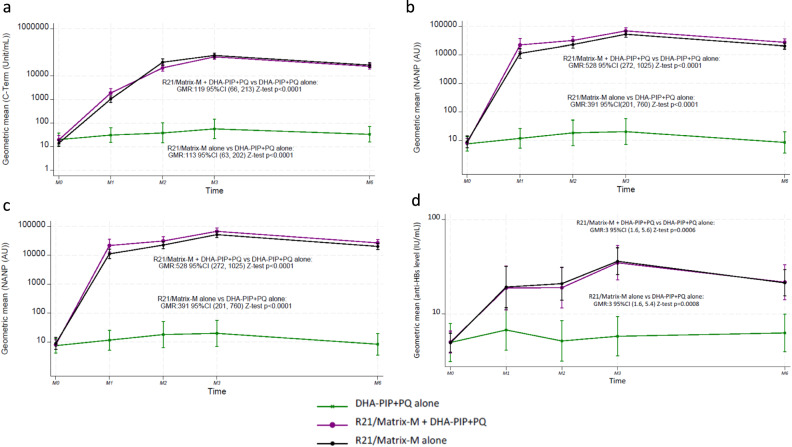
Serologic responses of adult Thai study participants vaccinated with the malaria vaccine R21/Matrix-M™ by study arm. **a** The line graph shows the geometric mean IgG concentration against the C-terminal end (C-term). The error bars indicate the 95% confidence intervals. **b** The line graph shows the geometric mean IgG concentration against six repeats of the central NANP6 region sequence (NANP6). **c** The line graph shows the geometric mean IgG concentration against full length R21. **d** The line graph shows the geometric mean of anti-HBs concentrations.

The geometric mean IgG concentrations against the three vaccine epitopes NANP6, C-Term, and full length R21 were similar when the vaccines were administered with ot without antimalarial drugs. The geometric mean concentrations of anti C-Term antibodies were significantly higher in Group 1 than Group 3 (GMR 119; 95%CI 66–213; Z-test *p* < 0.0001) and similarly higher in Group 2 than Group 3 (GMR 113; 95%CI 63–202; Z-test *p* < 0.0001; Fig. 3a). The geometric mean concentrations of anti NANP antibodies were significantly higher in Group 1 than Group 3 (GMR 528; 95%CI 272–1025; Z-test *p* < 0.0001) and also higher in Group 2 than Group 3 (GMR 391; 95%CI 201–760; Z-test *p* < 0.0001) as shown in Fig. 3b. The geometric mean concentrations of antibodies directed against full length R21 were significantly higher in Group 1 than Group 3 (GMR 116; 95%CI 68–196; Z-test *p* < 0.0001) and also higher in Group 2 than Group 3 (GMR 100; 95%CI 59–169; Z-test *p* < 0.0001; Fig. 3c). The geometric mean concentrations of anti HBs antibodies were overall orders of magnitude lower than antibodies against C-term or NANP but still significantly higher in Group 1 than Group 3 (GMR 3; 95%CI 1.6–5.6; Z-test *p* = 0.0006) and also higher in Group 2 than Group 3 (GMR 3; 95%CI 1.6–5.4; Z-test *p* = 0.0008; Fig. 3d).

The proportion of participants with a 4-fold increase in concentrations of C-Term and NANP6 at each timepoint compared to baseline were at least 98 (PP) and 90% (ITT) for either Group 1 or 2 at all time points (Table 6, Table S4). In comparison the proportion of participants with a 4-fold increase in concentrations of anti-HBs levels at each timepoint in Group 1 and 2 were at most 67%, they were still significantly higher in each of these arms compared to Group 3 alone at months 1, 2, 3 and 6 (Table 6). No difference in immunogenicity was observed between male and female participants (Supplementary Fig. 1a–d and Supplementary Table 5).

**Table 5:** Summary of participant visits

Group	Participants (*n*)	Visit time points														
		Scr.	M0				M1				M2				M3	M6
			D0	D1	D2	D7	D0	D1	D2	D7	D0	D1	D2	D7	D0	D0
1	50	x	x	x	x	x	x	x	x	x	x	x	x	x	x	x
2	50	x	x			x	x			x	x			x	x	x
3	20	x	x	x	x	x	x	x	x	x	x	x	x	x	x	x

Group 1 = R21/Matrix-M™ + DHA-PIP + SLD-PQ *n* = 50, Group 2 = R21/Matrix-M™ *n* = 50, Group 3 = No vaccine *n* = 20.

**Table 6:** Proportion of participants with a 4-fold or greater increase from baseline-PP analyses

Arm	Month 1 *n* (%)	Month 2 *n* (%)	Month 3 *n* (%)	Month 6 *n* (%)
C-Term				
Group 1	47/47 (100)	50/50 (100)	49/49 (100)	48/48 (100)
Group 2	48/49 (98)	48/48 (100)	48/48 (100)	48/48 (100)
Group 3	2/18 (11)	4/19 (21)	6/18 (33)	1/18 (6)
NANP6				
Group 1	50/50 (100)	50/50 (100)	50/50 (100)	48/48 (100)
Group 2	50/50 (100)	50/50 (100)	49/49 (100)	48/48 (100)
Group 3	2/20 (10)	4/20 (20)	5/20 (25)	2/20 (10)
Full length R21				
Group 1	45/45 (100)	48/48 (100)	48/48 (100)	46/46 (100)
Group 2	47/47 (100)	47/47 (100)	46/46 (100)	45/45 (100)
Group 3	6/18 (33)	3/18 (17)	6/18 (33)	3/16 (19)
Hepatitis B surface antigen				
Group 1	15/48 (31)	14/47 (30)	30/48 (63)	17/47 (36)
Group 2	13/49 (27)	20/49 (41)	33/49 (67)	18/48 (38)
Group 3	0/20 (0)	0/20 (0)	0/20 (0)	1/20 (5)

Group 1 = R21/Matrix-M™ + DHA-PIP + SLD-PQ *n* = 50, Group 2 = R21/Matrix-M™ *n* = 50, Group 3 = No vaccine *n* = 20; six repeats of the central NANP6 region sequence (NANP), C-terminal end (C-term), and the full-length vaccine (R21).

## Discussion

A combined malaria vaccine and mass drug administration strategy has the potential to accelerate falciparum malaria elimination in low transmission settings. The R21/MATRIX-M™ pre-erythrocytic malaria vaccine is a candidate for deployment in such a strategy. The vaccine was safe and well tolerated in combination with dihydroartemisinin-piperaquine-primaquine. There were no severe adverse events associated with R21/Matrix-M™ or the antimalarials. Participants receiving the vaccine combined with antimalarial drugs (Group 1) reported more unsolicited adverse events compared to participants in Group 2 receiving the vaccine only. These adverse events included a range of non-specific complaints. It is unlikely that the combination of R21/Matrix-M™ with antimalarial drugs triggers specific adverse events.

Reassuringly the pharmacokinetics of piperaquine was very similar when administered with or without R21/MATRIX-M™. There is no evidence of pharmacokinetic interactions between R21/Matrix-M™ and the antimalarial drugs used in the study.

R21/Matrix-M™ was highly immunogenic. Earlier experience of the co-administration of live, cell-culture rabies vaccines with the 4-aminoquinoline chloroquine resulted in reduced immunogenicity^7^. In contrast, the coadministration of R21/Matrix-M™ with piperaquine, a bisquinoline compound structurally related to chloroquine, did not have a negative impact on immunogenicity. The antibody concentrations against the C-term, NANP6 and anti-HBs were similar when administered with or without the antimalarial drugs. The concentrations of antibodies directed against the *P. falciparum* CSP antigens remained stable over the 6 months follow-up period. By contrast anti-HBs antibody concentrations were relatively low and showed signs of waning over the follow-up period. R21/Matrix-M™ contains a higher proportion of malaria to hepatitis B antigens than RTS,S/AS01 and appears to be correspondingly less effective as a hepatitis vaccine^8^.

Earlier studies conducted in 2018 also in the same centre in Bangkok demonstrated that the co-administration of RTS,S/AS01 with DHA/piperaquine and single low dose primaquine did not affect vaccine immunogenicity or piperaquine pharmacokinetics^9^. Taken together the previous and current studies strongly suggest that piperaquine does not affect the immunogenicity of the *Pf*CSP vaccines. Unfortunately, further field studies of RTS.S/AS01 in Asian populations could not proceed due to the unavailability of RTS,S/AS01. This highlights the problems of RTS,S/AS01 production capacity. By contrast R21/Matrix-M™ is produced by the large-scale manufacturer SIIPL which has a proven track record in high production capacity and managed during the COVID-19 pandemic to ramp up COVID-19 vaccine production over a short period to hundreds of million vaccine doses. In the absence of a head-to-head comparison, it is not possible to assess with certainty whether R21/Matrix-M™ is more immunogenic and by extension more protective than RTS,S/AS01.

The core strength of the study is the nearly complete follow-up of the first cohort of Asian R21/Matrix-M™ vaccinees in controlled circumstances. Nevertheless, the study is limited by a relatively small sample size. Infrequent adverse events cannot be detected without a very large sample. Second, we assessed only the pharmacokinetics of piperaquine and not the other antimalarials dihydroartemisinin and single low dose primaquine. Piperaquine is the partner drug that is responsible for eliminating residual parasites after the 3-day treatment to prevent recrudescence, and piperaquine concentrations at day 7 are highly associated with the risk of therapeutic success. It seems unlikely that the pharmacokinetics of dihydroartemisinin and primaquine is substantially influenced by co-administration with a vaccine but this cannot be excluded.

In conclusion, we found that R21/Matrix-M™, combined with and without antimalarial drugs, was safe, well-tolerated and immunogenic. This study opens the prospects for a large trial of mass vaccinations with R21/Matrix-M™ combined with mass antimalarial administration in Bangladesh (NCT06068530).

## Methods

This was a phase 2, open-label, computer-randomised, controlled safety, and immunogenicity trial of R21/Matrix-M™ in healthy, adult Thai participants conducted in the Clinical Therapeutics Unit, Faculty of Tropical Medicine, Mahidol University in Bangkok, Thailand between January and September 2023. The study protocol is included as a supplementary file (Supplementary Protocol).

### Study participants

After a full explanation of study procedures healthy Thai male or non-pregnant female participants, aged 18 to 55 years (inclusive), without a history of malaria, who provided informed consent and were willing to adhere to the study requirements were recruited. Participants were screened before enrolment for haematological or biochemical abnormalities, and for malaria and viral infections (HBsAg, HCV, HIV). A total of 120 participants who passed screening were randomised to one of three study groups and received three rounds of study drugs or vaccines one month apart:

- Group 1 (*n* = 50): 3 doses of R21/Matrix-M™ for three consecutive months concurrently with 3-days of daily dihydroartemisinin-piperaquine and a single low dose of primaquine (SLDPQ).
- Group 2 (*n* = 50): 3-daily doses of R21/Matrix-M™ alone for three consecutive months.
- Group 3 (*n* = 20): 3-days of dihydroartemisinin-piperaquine and SLDPQ for three consecutive months.

### Study vaccine/R21/Matrix-M™

R21 was manufactured by the SIIPL. Participants received a two-vial formulation: R21 was mixed immediately prior to administration with Matrix-M™, a saponin-based vaccine adjuvant (Novavax AB, Uppsala, Sweden). A dose of 10 μg R21 with 50 μg Matrix-M™ was used in the trial. All vaccines received three doses R21/Matrix-M™ one month apart, administered as a 0.75 ml dose intramuscularly into the deltoid muscle of the participant (Table 1). The amino acid residues incorporated in the R21 full and C-term constructs used in the ELISA are shown in Supplementary Methods.

### Antimalarial drugs

Dihydroartemisinin/piperaquine (DP) tablets (Shanghai Fosun Pharmaceutical Co., Ltd., China) for adult patients containing 40 mg dihydroartemisinin and 320 mg piperaquine were prescribed according to the participant’s bodyweight with a therapeutic dose range between 2 and 10 mg/kg/day dihydroartemisinin and 16–26 mg/kg/dose piperaquine. A single low dose of primaquine (0.25 mg base/kg; Government Pharmaceutical Organization GPO, Thailand) was co-administered on the first day of each round of antimalarials.

### Study procedures

Participants in treatment groups 1 and 2 received R21/Matrix-M™ on Day 0 of Month 0, 1, 2. Participants in treatment groups 1 and 3 received the standard DP treatment on Days 0, 1, 2, of Month 0, 1, 2. In addition, each participant in groups 1 and 3 received a single low dose of primaquine (0.25 mg base/ kg; Thai Government Pharmaceutical Organisation, Bangkok, Thailand) on the first day of each vaccination (Day 0). Local injection site and general solicited adverse events (AEs) were monitored on days 2, 3, and 7 post-vaccination. All other AEs (unsolicited) were recorded over a 28-day period after each vaccination. Serious AEs (SAEs) were captured throughout the study period. All injection site AEs were considered causally related to vaccination; the causality of all other AEs was assessed by the investigator. Haematological and biochemical tests for safety assessment were conducted at screening, on Day 0 and 7 of the first vaccination (M0) and Month 3. Abnormal test results were followed until they resolved.

### Pharmacokinetics

Plasma samples for quantification of piperaquine were collected during each vaccination round (Month 0, 1, and 2) on Day 0 (pre-1st dose), Day 1 (pre-2nd dose), Day 2 (pre-3rd dose), and Day 7 for group 1 and 3. Collected samples were stored at −80 °C and transferred to the Department of Clinical Pharmacology, Mahidol Oxford Research Unit, Bangkok, Thailand, for drug quantification. Piperaquine was quantified using liquid chromatography (LC) coupled with tandem mass spectrometry (MS/MS) detection according to a previously published and validated method^10^. The coefficient of variation for the quality control samples was <5% and the lower limit of quantification of piperaquine was set to 1.50 ng/mL. The impact of the vaccine dose on the observed drug concentrations of piperaquine were investigated by comparing drug concentrations in group 3 (standard vaccine dose) and group 1 using the Wilcoxon rank sum test. The pharmacokinetic comparison was made at each time point.

### Immunogenicity assessments

A validated four-plex enzyme-linked immunosorbent assay (ELISA) was used to measure IgG antibodies specific to four antigens. These comprised the full length R21 vaccine construct, six repeats of the central NANP6 amino acid repeat region of CS protein (NANP6), the C-terminus of CS protein (C-term), and Hepatitis B surface antigen (HBs). The amino acid residues incorporated in the R21 full and C-term constructs used in the ELISA are shown in the Supplementary Methods. This assay uses electrochemiluminescence (ECL) as a detection technique (Meso Scale Discovery (MSD, Rockville, Maryland, USA)). Samples were collected prior to first vaccination (M0), prior to second vaccination (M1D0), prior to third vaccination (M2D0), month 3 and month 6. The samples were processed at Jenner Institute, Oxford. Briefly, pre-coated malaria 4-plex plates (MSD, USA) are blocked for 30 min with casein (Thermo Fisher Scientific) at room temperature (RT) with shaking. After washing with PBS-Tween, plasma samples (diluted to between 1000 and 300,000 fold in casein (Thermo Fisher Scientific)) were plated in triplicate alongside pooled serum standards and QC samples. After incubation at RT with shaking for 2 h, plates were washed, and detection antibody SULFO-TAG Anti-human IgG (3D3cc; MSD, USA) was added for a further 1 h at RT with shaking. After washing, MSD GOLD read buffer B (MSD, USA) was added before the plate was read immediately using a MESO QuickPlex SQ 120 plate reader.

### Statistical consideration

Using the Blackwelder method for precision-based sample size calculations, a total of 20 participants per group was needed for group 3 to test against each of Groups 1 and 2 separately^11^. Using the Fisher’s exact test power simulations, a difference in serologic response of 30% (e.g., 50% vs 80%) resulted in 85% power with a sample size of 50 participants in each of Groups 1 and 2 testing at 5% significance level. Randomisation numbers were generated in blocks size 12, for the 3 study arms in a ratio of 5:5:2, for Group 1, Group 2, and Group 3 respectively.

All analyses were conducted according to a predefined Statistical Analysis Plan. Primary outcome analyses were carried out on both the according to per-protocol (PP) and the intention to treat (ITT) population. The PP analyses was the main strategy for the immunogenicity analyses. The safety outcomes were analyzed using the ITT approach. Participant demographic characteristics (age, gender, weight), and all other baseline information were summarised for each treatment group. The mean (SD) was used for continuous normally distributed data. The median (IQR) for continuous variables were used for skewed distribution variables. We approximated the total exposure to piperaquine in each treatment group by deriving the area under the plasma concentration time profile (AUC) for each individual up to the last measured drug concentration (AUC_0-d7_), using the cubic spline method for ascending concentrations and the logarithmic cubic spline method for descending concentrations. Observed drug concentrations at each time point was also compared between groups. Concentrations that were below the lower limit of quantification (LLOQ) were excluded from analysis only at the point when such values occurred. The raw immunology data were log-transformed using the natural log. The differences in the means between groups on log scale were calculated with the 95% confidence intervals for the differences in means. The differences in means on a log-scale and the corresponding 95% confidence intervals were back transformed by taking an exponential function. The resulting geometric mean ratios (GMR) and their 95% confidence intervals are reported. Statistical significance was declared as 5% level. Statistical analyses have been performed in Stata 18.0.

### Supplementary information

Supplementary Note

## Data Availability

Due to ethical and security considerations, the data that supports the findings in this study can be accessed only through the Data Access Committee at Mahidol Oxford Tropical Medicine Research Unit (MORU). The data sharing application form can be found here: https://www.tropmedres.ac/files/moru-bangkok-files/2-dataapplicationformv3-16nov2018.docx/view .
