# Supplementary material for: A randomised trial of malaria vaccine R21/Matrix-M™ with and without antimalarial drugs in Thai adults
Source: NPJ Vaccines. 2024 Jul 6;9:124. doi: 10.1038/s41541-024-00920-1 (PMC11227592; doi:10.1038/s41541-024-00920-1)
Supplement: Supplementary file 1 — Supplementary Note [file 41541_2024_920_MOESM1_ESM.pdf]

Supplementary Note to "A randomized, open label, trial to assess the safety, tolerability, and immunogenicity of the malaria vaccine, R21/Matrix-M™, combined with and without antimalarial drugs in Thai adults."

Table of contents:

- **Supplementary Methods:** the amino acid residues incorporated in the R21 full and C-term constructs used in the ELISA
- **Supplementary Figures:** Serologic responses by sex
- **Supplementary Table:** Serology GMR by sex
- **Study Note:** Study protocol

Supplementary Methods: the amino acid residues incorporated in the R21 full and C-term constructs used in the ELISA.

Full length R21 –

MDPNANPNANPNANPNANPNANPNANPNANPNANPNANPNANPNANPNANPNANPNANPNANPNANPNANPNANPNANPNANPNKNNQGNGQGHNMPNDPNR
NVDENANANSAVKNNNNEEPSDKHIKEYLNKIQNSLSTEWSPCSVTCGNGIQVRIKPGSANKPKDELDYANDIEKKICKMEKCSSVPVTNMENITSGFLGPLLVLQA
GFFLLTRILTIPQSLDSWWTSLNFLGGSPVCLGQNSQSPTSNHSPTSCPPICPGYRWMCLRRFIIFLFILLLCLIFLLVLLDYQGMLPVCPLIPGSTTTNTGPCKTCTTPA
QGNSMFPSCCCTKPTDGNCTCIPIPSSWAFAKYLWEWASVRFSWLSLLVPFVQWFVGLSPTVWLSAIWMMWYWGPSLYSIVSPFIPLLPIFFCLWVYI

C-term

CEPSDKHIKEYLNKIQNSLSTEWSPCSVTCGNGIQVRIKPGSANKPKDELDYANDIEKKICKMEKCSC

Supplementary Figures: Serologic responses of adult Thai study participants vaccinated with the malaria vaccine R21/Matrix-M™ by study arm. The line graphs are shown separately for malae and female study participants.

Supplementary Figure 1a The line graph shows the geometric mean IgG concentra�on against the C-terminal end (C-term). The error bars indicate the 95% confidence intervals.

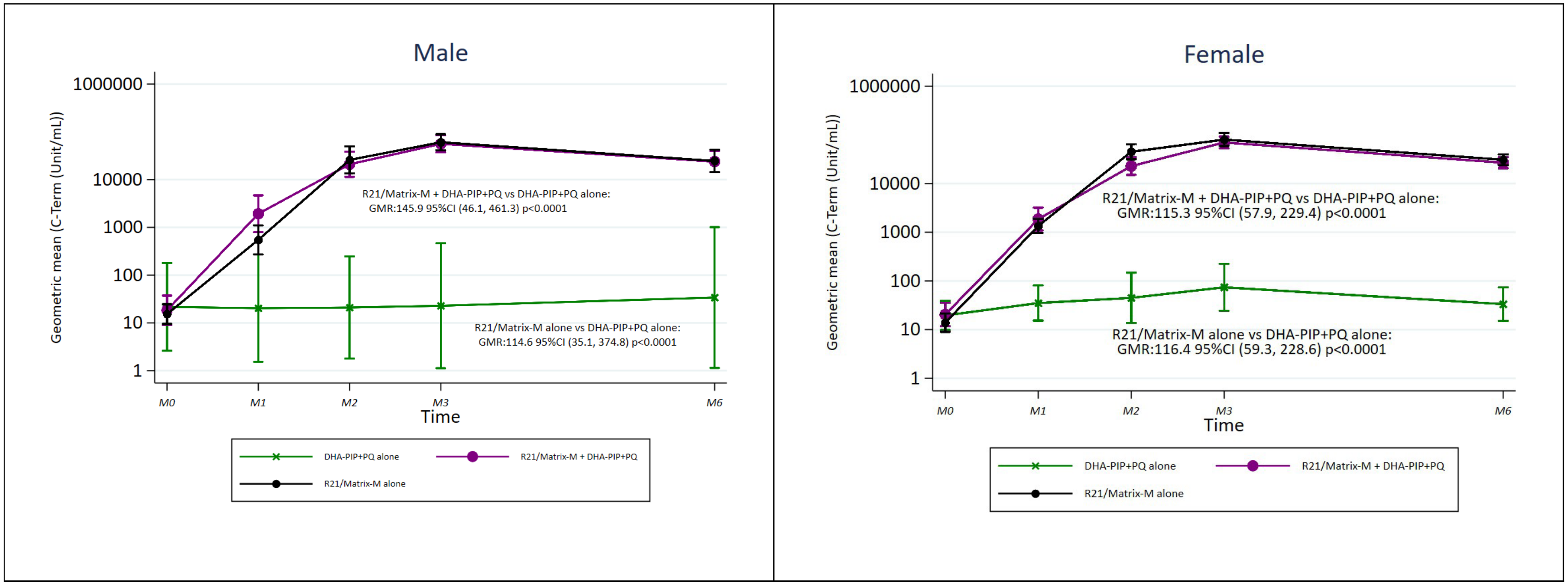

Supplementary Figure 1b The line graph shows the geometric mean IgG concentra�on against six repeats of the central NANP6 region sequence (NANP6). The error bars indicate the 95% confidence intervals.

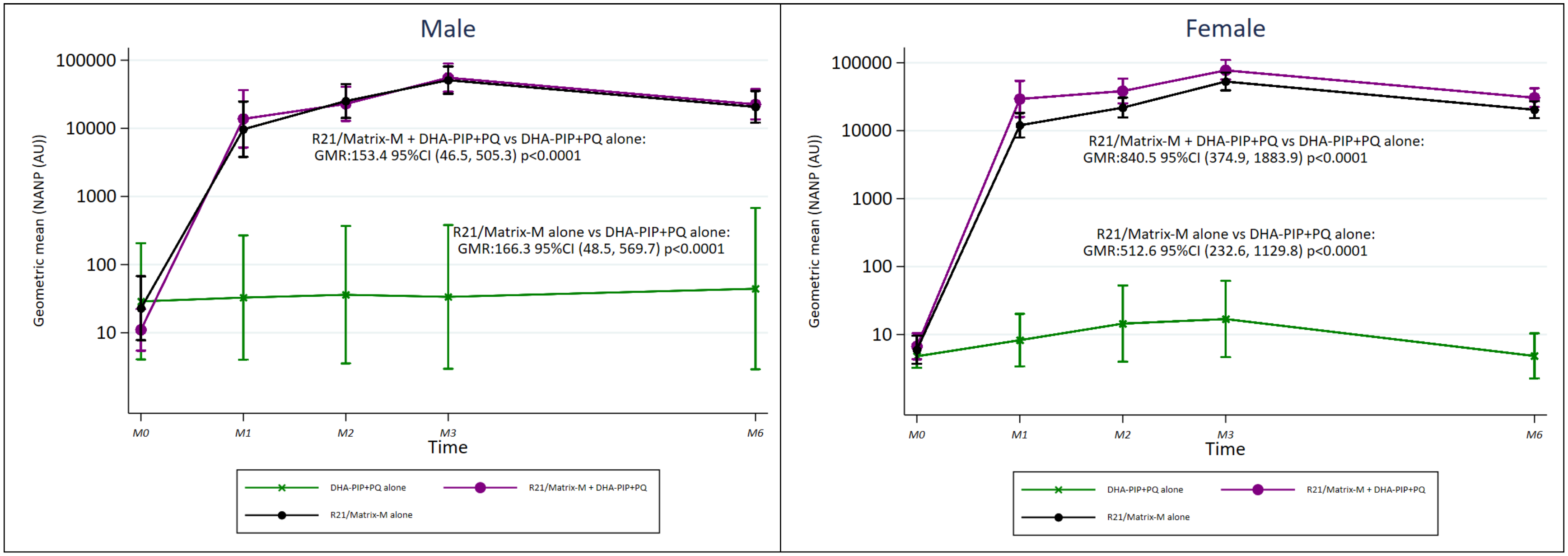

Supplementary Figure 1c The line graph shows the geometric mean IgG concentra�on against full length R21. The error bars indicate the 95% confidence intervals.

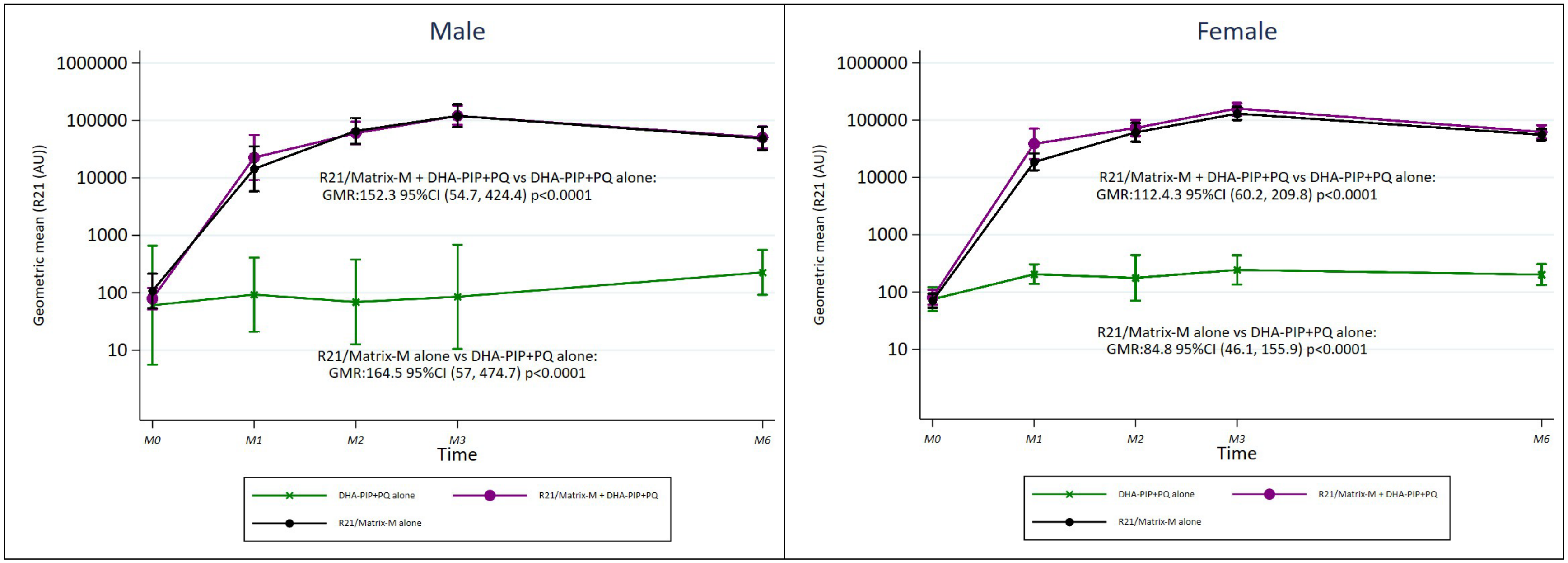

Supplementary Figure 1d The line graph shows the geometric mean of an�-HBs concentra�ons. The error bars indicate the 95% confidence intervals.

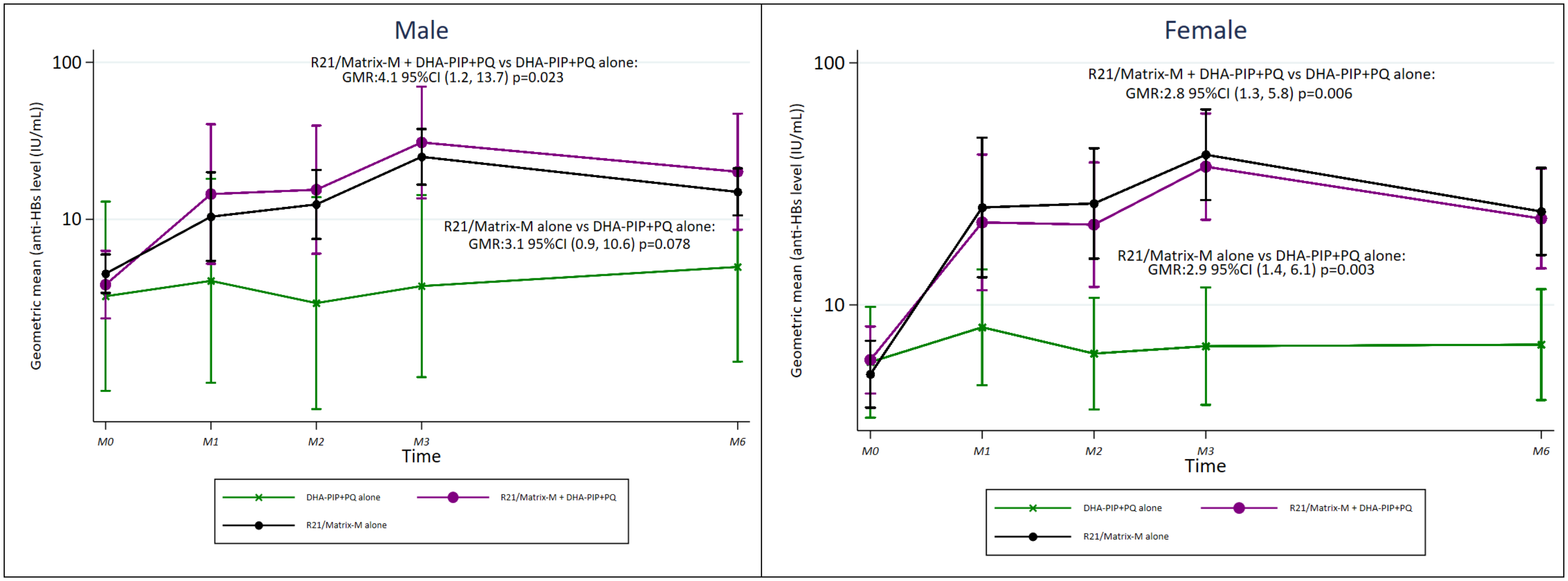

As shown in table Supplementary Table 1 there is no difference in immunological response between sex after adjusting for treatment/group. However, the proportion of males especially in the control group (DHA-PIP + SLD-PQ i.e. No vaccine) was very small i.e. 5 participants. Hence, this finding should be interpreted with a caution.

Supplementary Table 1 Analysis of immunology concentrations against sex adjusted for treatment/group

Parameter	GMR (95% CI)	P-value
C term: Male	Reference	
Female	1.3 (0.9, 2.1)	0.183
NANP: Male	Reference	
Female	0.8 (0.5, 1.4)	0.488
R21: Male	Reference	
Female	1.2 (0.8, 1.8)	0.388
Anti-HBs: Male	Reference	
Female	1.5 (0.9, 2.4)	0.084

A10.1 Full protocol summary

Malaria remains one of the leading causes of morbidity and mortality worldwide. *Plasmodium falciparum* is a complex pathogen with numerous immune evasion mechanisms which has delayed the development of safe and protective vaccines. There remains an urgent need to develop more protective and more affordable vaccine candidates that could achieve the World Health Organization (WHO) goal of 75% efficacy against clinical malaria.

R21/Matrix-M™ (R21/Matrix-M™) is a novel pre-erythrocytic candidate malaria vaccine targeting the pre-erythrocytic stages of *Plasmodium falciparum.* R21/Matrix-M™ can prevent progression to blood-stage disease, had a favorable safety profile and was well tolerated in earlier studies in adults and children. The majority of adverse events were mild, with the most common event being fever. None of the serious adverse events were attributed to the vaccine. At one year, vaccine efficacy in young African children remained high, at 77%. Currently there are no safety and immunogenicity data for the use of R21/Matrix-M™ in Asian populations. This trial will provide preliminary data to support a larger trial.

This study is a randomized, open-label, single centre, Phase 2 trial of R21/Matrix-M™. A total of **120 healthy non-pregnant Thai adults, aged 18-55 years**, inclusive, will be enrolled and randomised to one of three study groups:

- Arm 1: R21/Matrix-M™ in subjects who concurrently receive co-formulated dihydroartemisinin/piperaquine + single low dose of primaquine (SLDPQ).
- Arm 2: R21/Matrix-M™ alone.
- Arm 3: Co-formulated dihydroartemisinin/piperaquine + SLDPQ

We propose to conduct a safety and immunogenicity trial of R21/Matrix-M™. The major aims of this study are to: (1) assess the safety and immunogenicity of R21/Matrix-M™ in healthy Thai adults, (2) confirm that the co-administration of antimalarial drugs with R21/Matrix-M™ does not reduce the immunogenicity of the vaccine, and (3) assess the pharmacokinetics of antimalarial drug: piperaquine in subjects who concurrently receive R21/Matrix-M™. This study will contribute to our understanding of the immunogenicity of a vaccine, which could potentially be used to help stop the spread of malaria and anti-malarial resistance in the region.

B2. STUDY OBJECTIVES

B2.1 Primary Objective

- To assess the safety of the R21/Matrix-M™ vaccine, with and without concurrent anti-malarial administration, in Thai adults.

B2.2 Secondary Objectives

- To compare the immunologic response to R21/Matrix-M™ vaccine when concurrently given with DHA-PIP + SLD-PQ (Arm 1) with R21/Matrix-M™ vaccine alone (Arm 2) in Thai adults.
- To compare the piperaquine levels of participants who received the antimalarial drugs alone (Arm 3) with participants who received the antimalarial drugs combined vaccine (Arm 1).

B3. STUDY SITE(S)

Clinical study site:

This study will be conducted at the Clinical Therapeutics Unit (CTU), Hospital for Tropical Diseases, Faculty of Tropical Medicine, Mahidol University.

B4. STUDY TIMELINES:

We will recruit volunteers into this study after ethical approvals are granted from the Oxford Tropical Research Ethics Committee (OXTREC), and the Ethics Committee, Faculty of Tropical Medicine, Mahidol University. The volunteers will be included in this study for up to 7 months. We expect the recruitment period, data collection procedures, and to complete laboratory procedures and analysis approximately 18 months. The total duration for this study including preparations, regulatory approvals, and report writing is expected to be approximately 24 months.

B5. STUDY POPULATION AND SAMPLE

B5.1 Target population

One hundred and twenty healthy Thai adults aged 18 to 55 years, (inclusive) will be recruited into the study.

B5.2 Eligibility criteria

B5.2.1 Inclusion criteria

The participant is eligible to enter the study if all of the following apply:

1) Participant is a healthy adult, aged 18 to 55 years (both inclusive), of Thai origin.
2) Participant is willing and able to voluntarily give informed consent to participate in the trial
3) Able, in the investigator’s opinion, and willing to comply with the study requirements and follow-up.
4) Women of childbearing potential: must agree to practice continuous, effective contraception for the duration of the trial, and have a negative pregnancy test before each vaccination. (Costs for contraceptives will be reimbursed by the trial.)

B5.2.2 Exclusion criteria

The following criteria should be checked at the time of study entry. If ANY exclusion criterion applies, the subject must not be included in the study:

1) Pregnancy or breastfeeding, or planned pregnancy during the course of the study.
2) Presence of any medical condition (physical or mental) which, may place the participant at undue risk or interfere with the results of the study*. Including: serious cardiac, renal, hepatic, or neurological disease, severe malnutrition
3) Any confirmed or suspected immunosuppressive or immunodeficient condition. Including: history of splenectomy, human immunodeficiency virus (HIV) infection
4) Chronic administration (>14 days in total) of immunosuppressants or other immune-modifying drugs within six months of enrollment. Including: oral corticosteroids equivalent to prednisone > 20 mg/day[a]
5) History of an autoimmune disease
6) Hepatitis B surface antigen (HBsAg) or Hepatitis C antibody[b] detected in serum.
7) HIV antibody detected in serum
8) Screening electrocardiogram (ECG) demonstrating a QTc interval ≥ 450 ms
9) Finding on safety laboratory values as defined below:
   - AST > 2 x upper normal limit
   - ALT > 2 x upper normal limit
   - Anaemia (Hb < 10 g/dL),
   - Platelets < 150,000
   - Total bilirubin > 2 x upper normal limit
10) Abnormalities of examination or investigations at screening. Including: hepatomegaly, right upper quadrant abdominal pain or tenderness, abnormal blood tests (as defined in the protocol which are not listed above)
11) Positive malaria parasitaemia (RDT) at screening or baseline (Month 0, Day 0).
12) Receipt or planned receipt of an investigational medical product or participation in an interventional clinical trial during the study period
13) Contraindications to the use of artemisinins, piperaquine or primaquine*.
14) Use of medications with known potential interactions, prior allergic reactions to one or more components of the drug regimen.
15) History of allergic disease or reactions likely to be exacerbated by any component of the vaccine (e.g. egg products)
16) History of clinically significant contact dermatitis.
17) Contraindication to intramuscular (IM) injection*
18) Administration of a vaccine not included in the study protocol within 7 days of a study vaccine[c].
19) History of anaphylaxis post-vaccination.
20) Administration of immunoglobulins and/or any blood products during the period starting three months before the first dose of study vaccine or planned administration during the study period.

* subject to the investigator's judgement

Exceptions:

[a] Inhaled and topical steroids.

[b] Participation in hepatitis C vaccine study with confirmed negative HCV antibodies prior to participation in that study, and negative HCV RNA PCR at screening for this study

[c] The following vaccinations may be administered more than 7 days before or after a study vaccination: polio, diphtheria, tetanus, pertussis, hepatitis B, Haemophilus influenzae type b, Bacillus Calmette–Guérin (BCG vaccine), measles, influenza, pneumococcal disease, COVID-19 or yellow fever

B5.3 Sample size calculation

The sample size calculations are based on the objective of comparing the serologic response to R21/Matrix-M™ vaccine concurrently given with DHA-PIP + SLD-PQ with R21/Matrix-M™ vaccine alone in adults instead of basing it on safety outcome. This is a Phase II trial. Sample sizes for Phase II trials are usually small, as safety is still of paramount importance. Because of this limitation, these phase II studies are usually powered on the immunogenicity/efficacy endpoints because safety events are rare and a sample size based on safety would lead to a very high sample size that would not be acceptable from ethical standpoint. Another reason why powering it on immunogenicity/efficacy (a co-primary objective) is important, is that if the vaccine has poor immunogenicity profile then the safety question becomes redundant unless the study is a non-inferiority trial. We are optimistic that this sample size will be able to reveal important safety signals between arms which can be further explored in a phase III trial which would be a large trial that may be powered on safety endpoints that are rare. The efficacy endpoint (Immunogenicity objective) is a co-primary objective to the safety objective. We include both "safety" and "immunogenicity" terms in the study title because both of them are key endpoints**.** Valid sample sizes for comparing sample sizes between two groups are usually obtained using exact test methods such as the Fisher's exact test power calculations. This is done using simulations as sample size formulas are not well developed. To estimate the sample size we use a four-fold increase in anti –CS antibody titre as an assumed correlate of protection. For the sample size calculation, we assume that the background rate of response in the reference groups will be 5% and the true rate of anti-CS antibody response in the vaccine groups is 80%. We wish to exclude, for the vaccine, a one-tailed lower 95% CI for the reference-vaccine group difference of lower than 30%. The study aims to detect this difference with at least 90% power (because the detectable difference is big) and testing at 5% significance level. Based on these assumptions and using the method of Blackwelder for precision-based sample size calculations, a total of 20 participants per arm would be needed for arm 3 to test against each of arms 1 and 2 separately because the anticipated difference is too big. However, for arms 1 and 2 (Table 1 below), we will need more participants per group because a smaller detectable effect is anticipated. Using the Fisher's exact test power simulations, a difference in serologic response of 30% (say 50% vs 80%) gives more than 80% power (i.e. 85% power) with a sample size of 50 participants in each

of arms 1 and 2 testing at 5% significance level. Therefore, the total sample size for all the 3 arms will be 120 participants. The size of study groups is relatively small hence it is imperative to minimise potential losses. Whenever possible an attempt will be made to replace study participates should a participant be withdrawn or choose to withdraw. The stata command for obtaining the Fishers exact test power simulations is: power two proportions 0.50 0.80, test (fisher) n1(50) n2(50). This has been performed in Stata 16. The required sample sizes are summarized as follows:

Table 1. Sample size and vaccine vials required for each arm

Group	# Participants	Vaccine	Dose M0	Dose M1	Dose M2	Vaccine vials required
1	50	R21/Matrix-M™	Single standard dose (0.75mL)	Single standard dose (0.75mL)	Single standard dose (0.75mL)	300
2	50	R21/Matrix-M™	Single standard dose (0.75mL)	Single standard dose (0.75mL)	Single standard dose (0.75mL)	300
3	20	No vaccine	No vaccine	No vaccine	No vaccine	0
Total	120					600

Remark: R21/Matrix-M™ = adult formulation containing 10 µg of R21 and 50 µg Matrix-M in the standard dose of 0.75mL.

B5.4 Recruitment Methods

Volunteers will be recruited using methods previously successfully employed by the Clinical Therapeutics Unit (CTU) of the Mahidol-Oxford Tropical Medicine Research Unit. The volunteer unit maintains a register of candidates who expressed interest in the past to participate in clinical trials. In addition, volunteers will be recruited via flyers.

For Group 1, 10 volunteers will be enrolled into the study and receive the first vaccination.

Safety report will be submit to FTMEC. If there is no safety concern which related to the study procedure, then the recruitment of the remaining volunteers will be continued.

B5.5 Withdrawal/discontinuation criteria

Each participant has the right to withdraw from the study at any time and for any reason and is not obliged to give his or her reasons for doing so. Participants who are withdrawn because of AEs must be clearly distinguished from participants who are withdrawn for other reasons. For all withdrawals due to SAE/AEs, appropriate follow-up visits or medical care will be arranged, with the agreement of the participant, until the SAE/AE has resolved and stabilized

From an analysis perspective, a 'withdrawal' from the study is any participant who did not come back for the concluding visit or was not available for the concluding contact foreseen in the protocol.

A participant qualifies as a 'withdrawal' from the study when no study procedure has occurred, no follow-up has been performed and no further information has been collected for this participant from the date of withdrawal/last contact. Investigators will make attempts to contact those participants who do not return for scheduled visits or follow-up.

In addition, participant may be withdrawn from the study by the investigator at any time, if it is in the best interests of the participant's health and well-being, or for any of the following reasons:

- Ineligibility (either arising during the study or retrospectively, having been overlooked at screening).
- Significant protocol deviation.
- Participant demonstrates significant non-compliance with study requirements
- An AE, which requires discontinuation of the study involvement or results in inability to continue to comply with study procedures.

If a participant withdraws from the study, blood samples collected before their withdrawal from the trial will be used/stored unless the participant specifically requests otherwise. Data collected prior to the withdrawal of a participant will be used. In all cases of participant withdrawal or discontinuation, excepting those of complete consent withdrawal, long-term safety data collection will continue as appropriate if subjects have received one or more vaccine doses.

Participant withdrawal from the study agent

Participants withdrawn from the study agent are those who do not receive the complete number of vaccine doses. A participant withdrawn from the study agent may not necessarily be withdrawn from the study, as further procedures or follow-up may be performed (safety or immunogenicity), if planned in the study protocol. Information relative to premature discontinuation of the study agent will be documented in the CRF. The investigator will document whether the decision to discontinue further vaccination was made by the participant or the investigator and which of the following possible reasons was responsible for withdrawal:

- serious adverse event,
- non-serious adverse event,
- other (specify).

Subjects with positive malaria tests on vaccination days at Month 1 or Month 2 will be treated according to national guidelines and the volunteer will be withdrawn from further vaccination.

B5.6 Indications for deferral of vaccination

The following events constitute contraindications to administration of R21/Matrix-M™ at that point in time. If any one of these adverse events (AEs) occurs at the time scheduled for vaccination, the participant may be vaccinated at a later date, or withdrawn at the discretion of the investigator.

Acute disease at the time of vaccination (acute disease is defined as the presence of a moderate or severe illness with or without fever). All vaccines can be administered to persons with a minor illness such as diarrhoea, mild upper respiratory infection with or without low-grade febrile illness, i.e. Axillary temperature <37.5°C).

- Axillary temperature of ≥ 37.5°C at time of vaccination.
- Administration of a vaccine not foreseen by the study protocol within 7 days of any dose of R21/Matrix-M™ with the exception of vaccines against polio, diphtheria, tetanus, pertussis, hepatitis B, Haemophilus influenzae type b (Hib), Bacillus Calmette–Guérin (BCG vaccine), measles, influenza, pneumococcal disease, or yellow fever which may not be given within one week of vaccination.
- COVID-19 vaccines should not be administered 7 days before or after administration of the study vaccine due to potential adverse events e.g. fever.

B5.7 Absolute contraindications to further vaccination

The following adverse events (AEs) constitute absolute contraindications to further administration of R21/Matrix-M™. If any of these AEs occur during the study, the participant must not receive additional doses of R21/Matrix-M™ but may continue other study procedures at the discretion of the investigator. It is expected that the participant would continue full safety monitoring procedures, as per protocol.

- Acute allergic reaction or anaphylactic shock following the administration of vaccine investigational product.
- Pregnancy
- Any other findings that the investigator feels would increase the risk of having an adverse outcome from participation in the trial.

B5.8 Pregnancy

Participants are informed that the safety of the vaccine in pregnancy is unknown. Female study participants of childbearing potential are asked to use appropriate contraceptive methods to prevent pregnancy while they receive vaccinations. (Costs for contraceptives will be reimbursed by the study.) Appropriate contraceptive methods include:

- Established use of oral, injected or implanted hormonal contraceptives
- Intrauterine Device or Intrauterine System
- Barrier methods (condoms or diaphragm with additional spermicide)
- Male sterilisation of an exclusive partner (with appropriate post-vasectomy documentation of absence of sperm in the ejaculate)
- True abstinence, when this is in line with the preferred and usual lifestyle of the subject. Periodic abstinence (e.g., calendar, ovulation, symptothermal, post-ovulation methods) and withdrawal are not acceptable methods of contraception.

Female participants will be tested for pregnancy immediately prior to each vaccination.

Should a participant become pregnant during the trial, she will be followed up as other volunteers and in addition will be followed until pregnancy outcome, with the participant's permission. We will not routinely perform venepuncture on such participants.

B5.9 Multi-site research - N/A

B6. RESEARCH METHODOLOGY

B6.1 Details of study design

Summary of Trial Design

This study is a randomized, open-label, single centre, Phase 2 trial of R21/Matrix-M™ in healthy Thai adults. A total of 120 participants will be enrolled and randomized into one of three study arms in a ratio of 5:5:2 as described in Section 6.4.4. Each arm will have 50 participants (for arm 1 and 2) and 20 participants (for arm 3).

- For arm 1 and 3, the eligible volunteers will be asked to come into the Hospital for Tropical Diseases to receive the study agents as out-patient on Day 0 (the day of vaccination), Day 1, Day 2, and Day 7 of Study Months 0, 1 and 2; and on Day 0 of Study Months 3 and 6. There are a total of 15 study visits.
- For arm 2, the eligible volunteers will be asked to come into the hospital to receive the study agent (vaccination only) as out-patient on the day of vaccination (Day 0) and Day 7 of Study Months 0, 1 and 2; and on Day 0 of Study Months 3 and 6. There are a total of 9 study visits.

Table 2. Vaccine and drug administration

		Study period												
Timepoints	Month	Scr.	M0				M1				M2			
	Day of month		0	1	2	7	0	1	2	7	0	1	2	7
Interventions	Vaccination (Group 1 and Group 2)		x				x				x			
	DHA/piperaquine (Group 1 and Group 3)		x	x	x		x	x	x		x	x	x	
	PQ (single low dose primaquine) (Group 1 and Group 3)		x				x				x			

B6.2 Investigational Products:

1) R21/Matrix-M™

Description of Vaccine: The candidate R21 vaccine to be used has been developed by Jenner Institute and manufactured under cGMP conditions at Serum Institute India Private Limited (SIIPL). R21 is

produced to high levels in *Hansenula polymorpha* yeast in fermenters. The purified R21 antigen (drug substance) is released after testing, by SIIPL QC Department using qualified and validated methods. The quality released drug substance is used, for manufacturing drug product after appropriately diluting to 20 µg/mL using formulation buffer. The formulated blend is fill finished in siliconized glass vials (4 mL capacity) at a targeted concentration of not less than 20 µg/mL. Each vial is filled with 0.65 mL of the drug product to enable withdrawal of 0.5 mL. The final formulation has 20 mM Tris, 15% sucrose, 30 mM magnesium chloride, pH 7.4.

R21 will be used at a dose of 10 µg. The drug product is released after testing, by SIIPL QC Department using validated Test and Analysis Release Methods and certified by the Quality Assurance Department. The Quality Control Standards and Requirements for each candidate vaccine are described in separate Quality Assurance documents (e.g. release protocols, certificate of analysis).

Description of Matrix-M: Matrix-M (also known as Matrix-M1) is used as adjuvant for Malaria R21 vaccine. It contains purified saponin obtained from a crude extract of the plant Quillaja saponaria Molina; synthetic cholesterol and phosphatidyl choline (lipidic constituents of eggs). Both cholesterol and phosphatidylcholine are ubiquitous human cellular components and are not known to be allergens. Its saponin content is a mix of 85% Matrix-A and 15% Matrix-C w/w ratio. Matrix-M adjuvant GMP bulk is manufactured by Novavax AB and provided to SIIPL, at a saponin concentration of 1 mg/mL. The bulk was released after testing, by SIIPL QC Department.

Matrix-M adjuvant has been used in vaccines against RSV, influenza and most recently, extensively in COVID-19 vaccine trials. A COVID-19 vaccine with Matrix-M as an adjuvant (50µg dose) is currently undergoing rolling review for approval (approved for use in the UK) and licensure after successful Phase III trials enrolling many thousands of vaccinees. In the Phase III trial currently underway in Africa, more than 9000 doses of R21/Matrix-M have been safely administered to children aged 5-36 months without any major safety concerns (Unpublished data) across Kenya, Tanzania, Mali and Burkina Faso. For manufacturing of adjuvant vials, the Matrix-M bulk is fill finished at 200 µg/mL in siliconized glass vials (4 mL capacity). The Matrix-M is formulated in formulation buffer (1 mM KCl, 7.3 mM Na2HPO4, 2.5 mM KH2PO4 and 50 mM NaCl, pH 7.2) and stored at 2-8 °C until further use. The adjuvant vials are released after testing, by SIIPL QC Department, with a final certification from Quality Assurance Department. Matrix-M will be used at a dose of 50µg

The mixing prior to administration strategy will involve withdrawal of 0.5 mL antigen from one vial of R21 Malaria vaccine and adding it to Matrix-M1 vial. 0.5 mL of R21 antigen shall be withdrawn from another vial of R21 Malaria vaccine and be added to the same Matrix-M1 vial. The total volume in Matrix-M1 vial will be 1.5 mL. After addition the content will be mixed gently, and 0.75 mL of

the mixture will be withdrawn and administered to participants. Each dose of 0.75mL (after mixing of R21 with Matrix-M1) will contain 10 µg of R21 and 50 µg of Matrix-M1.

<u>Administration of Vaccine</u>: Vaccine preparation will be undertaken under aseptic conditions and in accordance with the trial SOPs. Vaccination administration and vaccine handling will be performed according to the relevant SOPs. The study participants will be observed closely for at least 30 minutes following the administration of study vaccine, with appropriate medical treatment readily available in case of an anaphylactic reaction.

2) Anti-malarial therapy

Participants in Arms 1 and 3 will receive three rounds of antimalarial drugs - each round starting on the day of vaccination (Day 0) at Study Months 0, 1, and 2. Each round consists of three daily doses of co-formulated dihydroartemisinin/piperaquine on Day 0, 1, and 2 (i.e. the day of vaccination and each day for 2 days after). Dihydroartemisinin/piperaquine tablets (Guilin Pharmaceutical Company or Sigma Tau Pharmaceuticals) for adult patients each contain 40 mg dihydroartemisinin and 320 mg piperaquine with a therapeutic dose range between 2–10 mg/kg/day dihydroartemisinin and 16–26 mg/kg/dose piperaquine (Table 3). In addition, each participant will receive a single low dose primaquine on the day of vaccination (Day 0) (Table 4). One single low dose primaquine of approximately 0.25 mg/kg (Thai Government Pharmaceutical Organisation) will be administered. This drug regimen has been used in mass drug administrations in more than 10,000 participants which have established that this antimalarial drug regimen is safe and clears gametocytes(1).

In the unexpected event that volunteers are found to be infected with malaria during screening, baseline or during follow-up examination they will be treated according to the most up-to-date government guidelines.

Table 3. DHA-PIP dosing schedule

	One tablet contains 40mg DHA and 320 mg PIP		
Bodyweight in kilograms	Number of tablets/day	Total tablets/month	Total tablets/study
41 - 50	2.5	7.5	22.5
51 - 60	3	9	27.0
61 - 70	3.5	10.5	31.5
71 - 84	4	12	36.0
85 - 100	5	15	45.0

Table 4. Primaquine dosing schedule

	One tablet contains 15mg primaquine		
Bodyweight in kilograms	Number of tablets/day	Total tablets/month	Total tablets/study
41 - 50	0.5	0.5	1.5
51 - 60	1.0	1.0	3.0

61 - 70	1.0	1.0	3.0
71 - 84	1.5	1.5	4.5
85 - 100	2.0	2.0	6.0

B6.2.1 Storage of vaccines and anti-malarial medications

The R21 vaccine antigen and Matrix-M adjuvant must be stored according to the manufacturer's recommendations at +2°C to +8°C. Any temperature excursions outside the range +2°C to 8°C must be reported to the Jenner Institute and SIIPL as soon as detected. Following an exposure to such a temperature deviation, vaccines will not be used until approval has been given by the study PI in consultation with Jenner Institute and SIIPL. Study anti-malarial medications must be stored at room temperature below 30 degrees Celsius. All vaccines and study medications will be stored in a safe and locked place, with no access to unauthorized personnel. Only trained, authorized study staff will have access to the vaccines and study medications. Storage temperatures will be monitored daily, according to standard operating procedures (SOPs) at the investigator's site. In addition, for vaccine storage refrigerator(s), an alarm system and a back-up refrigerator will be available in case of power failure/breakdown. All movements of the study vaccines will be documented and cold boxes, with temperature monitoring, will be used to transfer vaccines to the area where it will be administered. Vaccine and study medication accountability, storage, and handling will be in accordance with local SOPs. This study will not be blinded; i.e. it is an open-label study.

B6.2.2 Accountability of the study agents

The study site will maintain a record of the numbers of all vaccines and DHA/PIP + primaquine tablets obtained, used by the participants and returned to the pharmacy room in CTU. On study completion, a copy of the drug accountability record will be filed in the study folder.

B6.2.3 Concomitant medications

There will be no restrictions on concomitant medication beyond the exclusion criteria listed above.

B6.3 Study Endpoints

B6.3.1 Primary Endpoints

The safety of the investigational vaccine in each group:

- Occurrence of solicited from the date of each vaccination to 7 days of each vaccination.
- Occurrence of unsolicited adverse events (AEs) from the date of the first vaccination to 28 days after the last vaccination, according to the MedDRA classification.
- Occurrence of serious adverse events (SAEs) during the whole study period, i.e. during a 6-month follow up period from the receipt of first vaccination, according to the MedDRA classification.

B6.3.2 Secondary Endpoints

- For Arms 1 and 2, the concentration of antibodies against *Plasmodium falciparum* circumsporozoite (anti-NANP total IgG antibody), one month after the first dose (at Study month 1), one month after the second dose (at Study month 2), one month after the third dose (at Study Month 3) and six months after the first dose (at Study Month 6).
- Exploratory immunology endpoints including but not limited to cellular immunity.
- For Arms 1 and 3, piperaquine levels following the administration of the antimalarials with or without vaccine. (timepoints)

B6.4 Details of procedures for specimen/data collection

Procedures will be performed at the time points indicated in the schedule of procedures (Table 5). Additional procedures or laboratory tests may be performed, at the discretion of the investigators if clinically necessary (e.g. follow up repeat haematology or biochemistry laboratory tests in the event of clinically significant abnormal results).

Table 5. Study Procedures

		Study period														
Timepoints	Month	Scr.	M0				M1				M2				M3	M6[a]
	Day of month		0[c]	1	2	7	0	1	2	7	0	1	2	7	0	0
	Informed Consent	x														
	Eligibility screen	x														
	Inclusion / Exclusion criteria	x	x				x				x					
	Enrolment		x													
	Allocation		x													
Interventions	Vaccination (Group 1 and Group 2)		x				x				x					
Interventions	DHA/piperaquine (Group 1 and Group 3)		x	x	x		x	x	x		x	x	x			
Interventions	PQ (single low dose primaquine) (Group 1 and Group 3)		x				x				x					
Assessments	History of fever, current axillary temperature*	x	x	x	x	x	x	x	x	x	x	x	x	x	x	x
Assessments	Full medical history, vital signs, physical examination	x	x				x				x					
Assessments	Ask about any concomitant medication	x	x	x	x	x	x	x	x	x	x	x	x	x	x	x
Assessments	Assessment of any adverse event(s)		x	x	x	x	x	x	x	x	x	x	x	x	x	x
Assessments	Assess for any withdrawal criteria		x	x	x	x	x	x	x	x	x	x	x	x	x	x
Assessments – Blood sampling for laboratory assessments	Blood tests	Unit (mL)														
Assessments – Blood sampling for laboratory assessments	*P. falciparum* test**	2	2				0.5				0.5				2	0.5
Assessments – Blood sampling for laboratory assessments	CBC					2				2				2		
Assessments – Blood sampling for laboratory assessments	Biochemistry ALT, AST, CREA, BUN, Bilirubin	3	3			3				3				3	3	
Assessments – Blood sampling for laboratory assessments	HBsAg, HCV, HIV	1														
Assessments – Blood sampling for laboratory assessments	Immunology panel [b]		20				20				20				20	20
Assessments – Blood sampling for laboratory assessments	HLA typing	10														
Assessments – Blood sampling for laboratory assessments	Piperaquine levels***		1	1	1	1	1	1	1	1	1	1	1	1		
Assessments – Blood sampling for laboratory assessments	Blood volume per visit (mL)	16	26	1	1	6	21.5	1	1	6	21.5	1	1	6	25	20.5
Assessments – Blood sampling for laboratory assessments	Maximum cumulative blood volume (mL)	154.5														

	Electrocardiogram (ECG)	x														
	Urine pregnancy test	x	x				x				x					
Diary card provided			x				x				x					
Diary card return						x				x				x		

* Axillary temperature will be recorded from D0-D7 visits after each vaccination. If the symptoms are indicated to COVID-19, COVID-19 test will be done. If COVID-19 positive is indicated, then hospital guideline for COVID-19 case management will be followed. Study team will make decision whether or not to withdraw volunteer from the study case by case.

** *P. falciparum* test for purposes of indicating whether volunteer has been exposed, and not as an assessment of protection.

*** Piperaquine levels for group 1 and 3 only. Additional drug level assessment for Primaquine will be done, if additional data are required. Blood sample will be collected before each dose (at each visit) and then at day 7 for drug levels assessment.

a. first day of M6
b. the immunology panel consists of assays to be processed at UK laboratories and may include:
 - Serology total IgG, IgG avidity, subclasses and isotypes against full length R21, CS C-term, NANP and HBsAb.
 - Functional assays to measure antibody function
 - Flow cytometry assays effector and memory T cells (e.g. CD4+ and CD8+), T follicular helper cells, regulatory T cells, B cells, plasma cells and dendritic cells
 - Enumeration of antibody-secreting cells (e.g. B and plasma cells)
 - Antibodies against viral pathogens including cytomegalovirus.
 - Other ELISA assays for immunity to malaria.
c. If interval between screening and M0D0 is 7 days or less, screening lab results (haematology, biochemistry, *P. falciparum* test, and urine pregnancy test) can be used for enrolment (M0D0).

Table 6. Summary of participant visits

Group	Number of visit	Visit time points														
		Scr.	M0				M1				M2				M3	M6
			D0	D1	D2	D7	D0	D1	D2	D7	D0	D1	D2	D7	D0	D0
1	15	x	x	x	x	x	x	x	x	x	x	x	x	x	x	x
2	9	x	x			x	x			x	x			x	x	x
3	15	x	x	x	x	x	x	x	x	x	x	x	x	x	x	x

All the study procedures will be performed by the research team including research doctor, research nurse and research physician at the Clinical Therapeutics Unit at Faculty of Tropical Medicine, Mahidol University.

Table 7. Intervals between study visits

**Interval**	**Optimal length of interval**	**Allowed interval**
Screening to Month 0 / Day 0 (1st vaccination)	0 to 7 days	-
Month 0 / Day 0 (1st vaccination) to Month 1 / Day 0 (2nd vaccination)	28 days	+/ - 7 days

Month 1 / Day 0 (2nd vaccination) to Month 2 / Day 0 (3rd vaccination)	28 days	+/ - 7 days
Month 2 / Day 0 (3rd vaccination) to Month 3 / Day 0	28 days	+/ - 7 days
Month 3 / Day 0 to Month 6 / Day 0	84 days	+/ - 14 days

B6.4.1 Recruitment

Volunteers will be recruited using methods previously successfully employed by the Clinical Therapeutics Unit (CTU) of the Mahidol-Oxford Tropical Medicine Research Unit. The volunteer unit maintains a register of candidates who expressed interest in the past to participate in clinical trials. In addition, volunteers will be recruited via flyers.

B6.4.2 Screening and Eligibility Assessment (Screening Visit)

All potential volunteers will have a screening visit, which may take place up to 7 days prior to enrolment. Once informed consent is given, a screening number will be assigned in sequential order. Screening numbers will be issued consecutively (e.g. R21-001, R21-002, R21-003 etc.). This will be recorded in the Case Screening Form, together with the demographic information and procedures performed to determine eligibility. Screening procedures indicated in the schedule of procedures (Table 5) will be undertaken. If a screening haematology or biochemistry test result is out of the normal range, it may be repeated to ensure it is not a single occurrence. If an abnormal finding at screening is considered to be clinically significant, the volunteer will be informed, appropriate medical care arranged with the permission of the volunteer and the volunteer will be excluded from the study. In the unlikely event that a volunteer is found to have malaria or anaemia, the volunteer will be treated according to national guidelines and will be excluded from participating in the study.

B6.4.3 Enrolment and Baseline Assessment

All inclusion and exclusion criteria will be checked before enrolment in the study. Physical examination will be performed. Any new medical issues or symptoms that have arisen will be assessed. Blood will be collected for baseline *P. falciparum* test, clinical hematology and biochemistry. Blood will be collected and stored for measurement of antibodies against *P. falciparum* circumsporozoite (anti-CS antibody) until shipment to the reference laboratory. Urine will be collected from women of child-bearing potential for immediate pregnancy test.

If the interval between screening and enrolment is 7 days or less, the screening test results (*P. falciparum* test, haemoglobin, biochemistry, and urine pregnancy test) can be used for enrolment evaluation. In such cases, the tests (*P. falciparum* test, clinical hematology, biochemistry, and urine pregnancy test) will not be repeated at the baseline (Month 0 / Day 0) visit.

If all inclusion criteria are fulfilled and none of the exclusion criteria apply, the patient will be enrolled into the study and a case record form (CRF) specific to each participant completed. Regimen

allocation and administration of the vaccine will be on Day 0 Month 0. The randomization lists will be prepared by MORU statistician based on random numbers generated in STATA. The randomization sealed envelopes will be prepared by Clinical Trials Support Group (CTSG).

B6.4.4 Randomisation

Randomization numbers will be generated in blocks, for the 3 study arms in a ratio of 5:5:2, as follows:

- R21/Matrix-M™ + DHA-PIP+PQ (Arm 1)
- R21/Matrix-M™ alone (Arm 2)
- DHA-PIP+PQ alone (Arm 3)

Study participants will be assigned the next available randomization number on the list, and thus will be randomly allocated to Group 1, 2, or 3. This is an open-label study. Participants and clinical investigators will not be blinded to group allocation.

B6.4.5 First vaccination or anti-malarial medications initiation (Month 0 / Day 0 visit)

Participants in Group 1 and 2 will be vaccinated by intramuscular (IM) needle injection into the deltoid region of the arm. Participants in Group 1 will also receive anti-malarial medications. Participants in group 3 will receive anti-malarial medications only.

The study participants will be observed closely for at least 30 minutes following the administration of each study vaccine, with appropriate medical treatment readily available in case of an anaphylactic reaction.

An oral thermometer, tape measure and a paper diary card for solicited AEs will be given to each participant along with the emergency 24-hour telephone number to contact the on-call study physician if needed. Diary cards will collect information on the timing and severity of the following solicited AEs (Table 8):

Table 8. Solicited AEs

Local solicited AEs	Systemic solicited AEs
Pain at injection site	Fever
Redness at injection site	Diarrhoea
Swelling at injection site	Nausea
Bruising at injection site	Vomiting
	Fatigue
	Headache
	Muscle pain (Myalgia)
	Joint pain (Arthralgia)
	Chills

Participants will be instructed on how to self-assess the severity of these AEs. There will also be space on the diary card to self-document unsolicited AEs, and whether medication was taken to relieve the symptoms.

B6.4.6 Subsequent vaccination (, or subsequence rounds of anti-malarial medications) visits (Month 1 / Day 0 and Month 2 / Day 0 visits)

Subsequent vaccination visits will take place according to the schedule of procedures (Table 5. Physical examination will be performed. Any new medical issues or symptoms that have arisen since the last visit will be assessed. Blood will be collected for *P. falciparum* test. Participants with parasitaemia, or anaemia will be treated according to national guidelines. Blood will be collected and serum stored for measurement of antibodies against *P. falciparum* circumsporozoite (anti-CS antibody) until shipment to the reference laboratory. Urine will be collected from women of child-bearing age for immediate pregnancy test prior to administration of the investigational treatment.

B6.4.7 Follow-up Assessments

Follow-up assessments (visits not involving vaccination) will take place according to the schedule of procedures (Table 5). Information will be recorded in the CRF for follow-up visits. All participants will be reviewed according to the schedule, either by attending the clinic or being visited at home by a health visitor.

Diary cards will be reviewed for details of solicited and unsolicited AEs, and any new or undocumented medical issues or symptoms that have arisen will also be assessed and recorded in the adverse event CRF. Further clinical assessment in the form of detailed history and physical examination will be undertaken if appropriate.

B6.5 Schematic diagram of study design, procedures and stages, step-by-step

Obtaining approval from the Ethics Committee
↓
Recruiting research participants
↓
Inform consent process
↓
Screening and eligibility assessment
↓
Vaccination / drug administration
↓
Data collection
↓
Sample analysis
↓
Data analysis

B6.6 Specimen and data management

B6.6.1 Collecting and sample handling process (Blood tests)

Blood will be drawn at the time points indicated in the schedule of procedures (Table 5.) and the following laboratory assays performed:

At screening:

- Complete Blood Count (CBC)
- Biochemistry: alanine aminotransferase (ALT), aspartate aminotransferase (AST), total bilirubin, and BUN, creatinine (CREA).
- Diagnostic serology: HBsAg, HCV antibodies, HIV antibodies (Counselling will be given prior to testing blood for these blood-borne viruses.)
- *P. falciparum* test*
- HLA typing

Allocation and post-allocation

At M0, D0: *P. falciparum* test*, CBC, Biochemistry (ALT, AST, CREA, BUN, Bilirubin), anti-CS antibody; Pharmacokinetics** (piperaquine and primaquine drug levels will be assessed if additional data are required)
At M0, D1: Pharmacokinetics** (piperaquine and primaquine drug levels)
At M0, D2: Pharmacokinetics** (piperaquine drug levels)

At M0, D7: Haematology: Hb, WBC, PLT; Biochemistry: ALT, AST, BUN, Bilirubin, CREA; Pharmacokinetics**
At M1, D0: *P. falciparum* test*, anti-CS antibody; Pharmacokinetics**
At M1, D1: Pharmacokinetics**
At M1, D2: Pharmacokinetics**
At M1, D7: Haematology: Hb, WBC, PLT; Biochemistry: ALT, AST, BUN, Bilirubin, CREA; Pharmacokinetics**
At M2, D0: *P. falciparum* test*, anti-CS antibody; Pharmacokinetics**
At M2, D1: Pharmacokinetics**
At M2, D2: Pharmacokinetics**
At M2, D7: Haematology: Hb, WBC, PLT; Biochemistry: ALT, AST, BUN, Bilirubin, CREA; Pharmacokinetics**
At M3: *P. falciparum* test*, Haematology: Hb, WBC, PLT; Biochemistry: ALT, AST, BUN, Bilirubin, CREA; anti-CS antibody
At M6: *P. falciparum* test*, anti-CS antibody

* In the unexpected event that volunteers are found to be parasitaemic during screening, baseline or during vaccination or follow-up visits, they will be treated according to government guidelines. If at screening or baseline, these volunteers will be excluded from participation in the study. If at Month 1 or Month 2 vaccination days, these volunteers will be withdrawn from further study vaccination.
** Samples for pharmacokinetics will only be taken for subjects in Groups 1 and 3. Additional drug level assessment for primaquine will be done if additional data are required

Samples collected for screening assays including CBC, biochemistry, diagnostic serology and *P. falciparum* test will be tested in Bangkok at Hospital for Tropical Disease Laboratory, Faculty of Tropical Medicine, Mahidol University.

Samples collected for drug levels assessment will be tested at the Department of Clinical Pharmacology Laboratories, MORU, Faculty of Tropical Medicine, Mahidol University.

Samples collected for HLA typing and assessment of immunologic responses will be processed at the site, and stored frozen until shipment to the Jenner Institute, Oxford UK for testing.

The following investigations will be done on blood samples collected for immunogenicity endpoints and exploratory immunology, at the discretion of the investigators:
- Serology to quantify and characterise the binding properties of antibodies to the vaccine components (full length R21, CS C-term, NANP and HBsAb), including total IgG, IgG avidity, subclasses and isotypes.
- Functional assays to measure antibody function, such as inhibition of sporozoites invasion and systems serology assays

- Flow cytometry assays to enumerate and functionally characterise immune cell populations such as effector and memory T cells (e.g. CD4+ and CD8+), T follicular helper cells, regulatory T cells, B cells, plasma cells and dendritic cells
- Enumeration of antibody-secreting cells (e.g. B and plasma cells)
- Assays to assess presence or absence of other factors affecting vaccine immunogenicity, such as antibodies against viral pathogens including cytomegalovirus.
- Other ELISA assays for immunity to malaria that may be relevant to prior malaria exposure and be used to predict vaccine immunogenicity.

Genetic tests for determination of Human Leucocyte Antigen (HLA-type) and associated genes that can have an impact on vaccination. N.B. Specific consent genetic testing will be sought through an additional question on the ICF to make clear to participants that consent for genetic testing does not affect participation in the clinical trial.

Drug levels

The study investigates two questions regarding the potential interactions between antimalarial drugs and vaccines.

1) Does the co-administration of antimalarial drugs with vaccines influence vaccine antibody levels
2) Does the co-administration of antimalarial drugs with vaccines influence drug levels.

The second question will be addressed for piperaquine only. Piperaquine levels will be assessed during each round (M0, M1, and M2) on Day 0, 1, 2, and 7 (at M0D0, drug level will be assessed, if additional data are required). (Table 9.) Additional drug level assessment for primaquine will be done if additional data are required.

Table 9. Pharmacokinetic blood sampling time.

Month(s)	Day(s)	PK*
M0	Day 0	x
	Day 1	x
	Day 2	x
	Day 7	x
M1	Day 0	x
	Day 1	x
	Day 2	x
	Day 7	x
M2	Day 0	x

Month(s)	Day(s)	PK*
	Day 1	x
	Day 2	x
	Day 7	x

Remark:

* Blood sample will be collected before each dose (at each visit) and then at day 7 for drug levels assessment. The drug levels will be assessed in the 50 participants who receive vaccine and drugs (groups 1) and 20 participants who receive drugs alone (group 3).

B6.6.2 Data Collection and Data Access

Source Data

Source documents are original documents, data, and records from which participants' CRF data are obtained. These include, but are not limited to, hospital records (from which medical history and previous and concurrent medication may be summarized into the CRF), clinical and office charts, registration logbook, laboratory and pharmacy records, diaries, radiographs, referral notes and correspondence. The study staff will derive data from source documents and record it into the volunteers' CRFs, which will be in paper and/or electronic format. This includes safety data, laboratory data (both clinical and immunological) and outcome data.

All documents will be stored safely in confidential conditions. On all study-specific documents, other than the signed consent, the participant will be referred to by a unique study participant number/code, not by name.

Direct access to source data/documents

Direct access to anonymised trial related source data/documents will be granted to authorized representatives from the sponsor, Jenner Institute, Research Ethics Committees (RECs), and the regulatory authorities to permit trial-related monitoring, audits and inspections.

Record Keeping

All study data will be stored under lock and key. The investigators will maintain and retain appropriate medical and research records and essential documents for this trial for a period of 5 years after completion of the study, while electronic data will be retained indefinitely. Only authorized, trained study staff will have access to study records. On all documents, other than the signed consent, the participant will be referred to by the study participant number, not by name.

The principal Investigator will be responsible for data management and for delegating the receiving, entering, cleaning, querying, analysing and storing all data that accrues from the study. Data will be managed and stored in MACRO® database, a GCP-compliant electronic data capture system. A study data management plan will outline detailed procedures for data capture, storage, curation and preservation.

The investigators will permit authorized representatives of the sponsor, ethical committee(s), regulatory agencies, authorized individuals from, Jenner Institute and the monitors to examine (and when required by applicable law, to copy) clinical records for the purposes of quality assurance reviews, audits and evaluation of the study safety and progress.

B6.7 Data analysis

B6.7.1 Study Endpoints

We will assess the R21/Matrix-M™ recipients with regards to:

Safety: the occurrence of solicited and unsolicited AEs and SAEs (please see safety section)

Immunogenicity: (a) serum anti-CS IgG antibody titres responses to NANP and anti-CS antibody avidity, (b) cellular immunity.

B6.7.2 Inclusion in Analysis

All patients who received at least one dose of vaccine will be included in the safety analyses. Patients lost to follow-up before the completion of the follow-up period assessments will be censored at the last day seen.

B6.7.3 Immunology analyses

Given the incidence of malaria in the study area, it is highly unlikely that any volunteer will become infected with any Plasmodium species during the trial. However, to exclude the remote possibility that a participant becomes infected we will screen participants for malaria at screening, Month 0, Month 1, Month 2, Month 3, and Month 6. Participants positive for malaria at screening or Month 0 are excluded from participation in the study and will not be enrolled or vaccinated.

B6.7.4 Overall plan

Both intention-to-treat and per-protocol analyses will be carried out. In the intention-to-treat analysis, every participant randomized in the study (who receive the correct or incorrect study agent, one or more doses, and complete or incomplete doses) will be analysed, except if he/she did not receive any dose of the study vaccine or if no post-randomization data was collected for this participant.

The per-protocol analysis will compare participants according to the study agent actually received and will include only those participants who satisfied the inclusion/exclusion criteria, followed the protocol, and received three complete, correct doses. The following non-compliant participants will be excluded:

- Participants included without meeting at least one inclusion criterion
- Participants included despite meeting at least one exclusion criterion
- Participants found non-compliant with the blood sampling schedule

- Participants vaccinated with the wrong study agent (non-compliance with the randomization code)
- Participants excluded from the intention-to-treat analysis.
- No interim analysis is planned.

B6.7.5 Analysis of baseline characteristics

The demographic characteristics of enrolled participants will be compared by study group.

B6.7.6 Analysis of immunogenicity

The serum anti-CS antibody titre and avidity

The main analyses will involve the serum anti-CS antibody titre and avidity. These parameters will be measured at baseline and then at follow-up times longitudinally. During statistical analysis, we will assess whether a participant had a titre that is greater than 4 times that of the participant's baseline titre. Then we will compute the number of participants who had a titre of greater than 4 times their baseline titres and obtain a proportion of these for each group. These proportions of participants with >4-fold rise in titre from baseline will be compared between arms at each follow-up time. A Fisher's exact test will be used to compare the proportions of participants with >4-fold rise in titre from baseline between groups at each follow-up. These proportions will also be summarised graphically by arm to visually assess the differences between arms over-time. This will help to visually assess whether time modifies the effect of group.

Furthermore, the absolute titre values will be analysed and compared between groups. Since the distribution of these titre values will likely be highly skewed overtime, these values will be log-transformed and compared between arms at the different follow-up times. In order to revert back to the original scale (taking exponentials), the Geometric Mean Ratios (GMR) of the titres will be the measures of effect of interest that will be used to compared the log-transformed titres between the arms. The 95% confidence intervals for the Geometric Mean Ratios will be presented. The mean titre trends (on log-scale) over time will also be plotted by arm to assess whether there is a difference in trends of the titres between arms over time.

Tests of significance will be performed at 5% significance level. Analyses will be performed in Stata version 17 or higher. A separate detailed Statistical Analysis plan will be developed, finalized and signed before databased lock.

B7. DATA AND SPECIMEN BANKING AND/OR SHARING

B7.1 Management of specimen/data archiving or remaining samples

With the volunteers' informed consent, any leftover serum will be frozen for future immunological analysis of vaccine- or malaria-specific responses. This may include human DNA and RNA analysis to search for correlates of vaccine immunogenicity and efficacy.

B7.2 Specimen/data sharing plan

All personal details of participants will be de-identified. These data including laboratory investigation results will be stored and may be shared with other researchers to apply in their research in accordance with the MORU data sharing policy.

PART C: ETHICAL CONSIDERATION (describe only the responsibilities of the PI)

C1. SIGNIFICANCE OF THE STUDY

This study's aims are to conduct a safety and immunogenicity trial of R21/Matrix-M™ in Thai adults. This trial will provide preliminary data to support a larger trial.

C2. BALANCE OF RISK AND BENEFIT

C2.1 Risk of the study

Phlebotomy

There may be minor bruising, local tenderness or pre-syncopal symptoms associated with venepuncture.

R21/Matrix-M™ vaccination

The most frequent adverse reactions observed in previous clinical trials using the R21/Matrix-M™ vaccine include pain, swelling, erythema, and tenderness at the site of injection, and systemic symptoms such as low-grade fever and short-term flu-like symptoms: fatigue, myalgia, headache, malaise. As with any vaccine, unexpected serious adverse events, including severe allergic reactions to the vaccine components, may occur, although this has not been reported in prior studies with R21/Matrix-M™ in adults. Potential immune-mediated disease (pIMD) is a theoretical concern with adjuvanted vaccines. A causal association between the adjuvant components and occurrence of autoimmune diseases has however not been established, as these can occur in people who get other vaccines, or no vaccines at all.

Antimalarial medications

Dihydroartemisinin/piperaquine (DHA-PIP)

DHA-PIP is the first line treatment for uncomplicated malaria in many malaria endemic countries in Asia and Africa and has an extraordinary robust safety profile (2-4). DHA-PIP is also very well tolerated. The main reported side effects are gastrointestinal upset (nausea, vomiting, abdominal pain and diarrhoea) as well as dizziness, headache and disturbed sleep. Rates of these side effects are generally < 10% and often <5% except for dizziness (~12%). There is no evidence that DHA- PIP can cause clinically significant QTc prolongation at therapeutic doses(5).

Primaquine (PQ)
Like all 8-aminoquinolones, primaquine is associated with haemolysis in G6PD deficient individuals in a dose-related relationship. To avoid any potential risk for haemolysis, participants will receive just a single low dose of primaquine (approximately 0.25mg/kg) on the day of vaccination, at each vaccine dose (for groups 1) and on Day 0 at each round of antimalarial drugs for three rounds (for group 3). This dose is safe even when administered to individuals with a genotype associated with severe G6PD deficiency(6). Other known side effects with primaquine administration include symptoms of gastritis and enteric complaints when administered for 14 days at large doses for the treatment of *Plasmodium vivax* malaria. Such abdominal complaints have not been observed when a single low dose primaquine is administered.

C2.2 Preventive and alleviative measures for risk

Phlebotomy
Participants' blood samples will be collected by well-trained medical personnel with aseptic technique to prevent infection or complications.

R21/Matrix-M™ vaccination
Volunteers will be vaccinated in a clinical area where Advanced Life Support trained physicians, equipment and drugs are immediately available for the management of any immediate post-vaccination serious adverse reactions. Participants will be observed closely for at least 30 minutes following administration of the vaccine. No SAEs attributable to the R21/Matrix-M™ were detected during the large trial in Burkina Faso.

Antimalarial medications
Dihydroartemisinin/piperaquine (DHA-PIP)
To exclude the possibility that pre-existing QTc prolongation results in AEs, the QTc of all volunteers will be measured and candidates with a QTc>450ms will not be enrolled.

COVID Risks for Study Participants
At the time of the drafting this document, it seems likely that the COVID-19 infections will be a background risk while the study is being conducted. The study will comply with the COVID-19 regulations and recommendations provided by the government, the university, and the hospital administration at the time of study implementation. The study relevant COVID-19 regulations will be included and updated in study operating procedures (SOPs).

C2.3 Benefits of the study
No direct benefit is foreseen from study participation. Meals to be provided to study participants at some study visits might be considered as a direct benefit and other possible indirect benefits may be possible since participants will be screened for human immunodeficiency virus (HIV), hepatitis B and C and will receive a medical check-up. Knowledge gained from this study will assist with the

development of a vaccine against *P. falciparum* malaria which could potentially be used to help stop the spread of malaria and anti-malarial resistance in the region.

C3. CONSIDERATION FOR VULNERABLE RESEARCH PARTICIPANTS

Check whether your study involves any of the following vulnerable research participants.

- ☐ Prisoners
- ☐ Pregnant women
- ☐ Mentally ill persons
- ☐ Cancer or terminally ill patients
- ☐ Neonates/infants/children (aged <18)
- ☐ HIV/AIDS patients
- ☐ Institutionalized persons e.g. military, students, etc.
- ☐ Others (please specify............

C4. INFORMED CONSENT ISSUES

C4.1. Informed consent process

The informed consent process: trained study personnel will explain the purpose of the study according to the information sheet prior to any study related procedures being undertaken. The participant must personally sign and date the latest approved version of the informed consent form before any study specific procedures are performed. In such cases where the participant is illiterate, informed consent will be conducted in the presence of an impartial witness who would sign and date the informed consent form, while the participant provides their thumbprint.

The information sheet and informed consent form will be explained to the participants detailing: the exact nature of the study; the implications and constraints of the protocol; the known side effects and any risks involved in taking part. The aims of the study and all tests to be carried out will be explained. It will be clearly stated that participation is entirely voluntary and that refusing to participate will not involve any penalty or affect the participants' right to receive standard medical care at the healthcare post. It will also be emphasized that if they do consent to participate and are enrolled, that they are free to withdraw from the study at any time, for any reason, without any penalty or prejudice to future care, and with no obligation to give the reason for withdrawal. The volunteers will have the opportunity to question the Investigator, or other independent parties to decide whether or not they will participate in the study.

Written informed consent will then be obtained by means of participant dated signature or thumbprint and witness dated signature, in the case of an illiterate participant, and dated signature of the person who presented and obtained the informed consent. The person who obtained the consent must be suitably qualified and experienced, and have been authorized to do so by the study site Principal Investigator. A copy of the signed Informed Consent and Information Sheet will be

given to the participants. The original signed form will be kept in the investigator's site file and retained at the study site.

Hospital consent for HIV test will be obtained prior to counselling and HIV test. Pre and post counselling for HIV screening, report and treatment for volunteer will be conducted with the support of certified staff from the Bangkok Hospital of Tropical Diseases. If a participant is found to be HIV positive, follow up measures including, but not limited to, providing counselling and treatment according to standard hospital procedure will be arranged through certified staff from the Hospital for Tropical Diseases.

C4.2 Informed consent documentation

Age	Informed Consent/ Assent Form	Participant Information Sheet
18 years and over (Adult)	ICF for Adult (v.4.0 dated 24 August 2022)	For adult (v.4.0 dated 24 August 2022)
	ICF for Data Sharing/Data and Leftover Specimen Storage from Current Study for Future Use v.4.0 dated 24 August 2022	
	ICF for Genetic Testing v.4.0 dated 24 August 2022	

C4.3 Compensation for research participants

☑ Yes, please provide details:

Reasonable compensation (e.g. for transport, parking, and lost working time) for any study visits will be reimbursed as appropriate. Subjects will receive compensation of 1,000 Thai Baht for each scheduled study visit.

Table 10. Participant compensation

Study visits	Group 1 and 3		Group 2	
screening	1,000 THB	30 USD	1,000 THB	30 USD
vaccination (Month 0 / day 0)	1,000 THB	30 USD	1,000 THB	30 USD
antimalarial day 2	1,000 THB	30 USD	NA	NA
antimalarial day 3	1,000 THB	30 USD	NA	NA
vaccination (Month 1 / day 0)	1,000 THB	30 USD	1,000 THB	30 USD
antimalarial day 2	1,000 THB	30 USD	NA	NA
antimalarial day 3	1,000 THB	30 USD	NA	NA

vaccination (Month 2 / day 0)	1,000 THB	30 USD	1,000 THB	30 USD
antimalarial day 2	1,000 THB	30 USD	NA	NA
antimalarial day 3	1,000 THB	30 USD	NA	NA
follow up (Month 0 / day 7)	1,000 THB	30 USD	1,000 THB	30 USD
follow up (Month 1 / day 7)	1,000 THB	30 USD	1,000 THB	30 USD
follow up (Month 2 / day 7)	1,000 THB	30 USD	1,000 THB	30 USD
follow up (Month 3 / day 0)	1,000 THB	30 USD	1,000 THB	30 USD
follow up (Month 6 / day 0)	1,000 THB	30 USD	1,000 THB	30 USD
total	15,000 THB	450 USD	9,000 THB	180 USD

☐ No, please provide reasons: ……………………………………………………..………

C4.4 Responsible and contact persons

☑ Person(s) responsible for payment for treatment of complications and adverse effects

☑ Person(s) including doctor(s) and/or contact address (es) and telephone number(s) for emergency use

1. **Dr. Podjanee Jittamala,** Department of Tropical Hygiene, Faculty of Tropical Medicine, Mahidol University, Tel. (02) 306-9157, Mobile (081) 956-3371.

2. **Dr. Borimas Hanboonkunupakarn**, Department of Clinical Tropical Medicine, Faculty of Tropical Medicine, Mahidol University, Tel. (02) 354-9100 ext. 3160, Mobile (086) 970-5705

3. **Kittiyod Poovorawan,** Department of Clinical Tropical Medicine, Faculty of Tropical Medicine, Mahidol University, Tel. (02) 354-9100 ext. 1435 Mobile (083) 149-6864

PART D: APPENDIX

APPENDIX A: REFERENCES

1. von Seidlein L, Peto TJ, Landier J, Nguyen TN, Tripura R, Phommasone K, et al. The impact of targeted malaria elimination with mass drug administrations on falciparum malaria in Southeast Asia: A cluster randomised trial. PLoS Med. 2019;16(2):e1002745.

2. Assefa DG, Yesmaw G, Makonnen E. Comparative effect of dihydroartemisinin-piperaquine versus artemether-lumefantrine on gametocyte clearance and hemoglobin recovery in children with uncomplicated Plasmodium falciparum malaria in Africa: a systematic review and meta-analysis of randomized control trials. Int J Infect Dis. 2021.

3. Assefa DG, Yismaw G, Makonnen E. Efficacy of dihydroartemisinin-piperaquine versus artemether-lumefantrine for the treatment of uncomplicated Plasmodium falciparum malaria among children in Africa: a systematic review and meta-analysis of randomized control trials. Malar J. 2021;20(1):340.

4. Assefa DG, Zeleke ED, Bekele D, Tesfahunei HA, Getachew E, Joseph M, et al. Efficacy and safety of dihydroartemisinin-piperaquine versus artemether-lumefantrine for treatment of uncomplicated Plasmodium falciparum malaria in Ugandan children: a systematic review and meta-analysis of randomized control trials. Malar J. 2021;20(1):174.

5. Wang Q, Zou Y, Pan Z, Zhang H, Deng C, Yuan Y, et al. Efficacy and Safety of Artemisinin-Piperaquine for the Treatment of Uncomplicated Malaria: A Systematic Review. Front Pharmacol. 2020;11:562363.

6. Eziefula AC, Pett H, Grignard L, Opus S, Kiggundu M, Kamya MR, et al. Glucose-6-phosphate dehydrogenase status and risk of hemolysis in Plasmodium falciparum-infected African children receiving single-dose primaquine. Antimicrob Agents Chemother. 2014;58(8):4971-3.

APPENDIX B: SAFETY TERMS AND ADMINISTRATIVE INFORMATION

1. **SAFETY ASSESSMENT AND REPORTING**

1.1 Safety reporting

The investigator or site staff is/are responsible for the detection, documentation and reporting of events meeting the criteria and definition of an AE or SAE as provided in this protocol. Each subject will be instructed to contact the investigator immediately should the subject manifest any signs or symptoms they perceive as serious.

Reporting procedures for serious AEs

In order to comply with current regulations on serious adverse event reporting to regulatory authorities, the event will be documented accurately and notification deadlines respected. SAEs will be reported to a safety group (specified members of the study team) and medical monitor immediately (within 24 hours) of the investigators being aware of their occurrence. This safety group includes the Principal Investigator, who acts on behalf of the sponsor for notification of SAEs. SAEs will also be reported to ethics committees, the regulatory authority, and – in subjects who have received vaccine – to the manufacturer of the adjuvant (Novavax) in accordance with reporting requirements and according to required timelines in safety data exchange agreement(s).

1.2 Safety definitions

1.2.1 Definition of an adverse event

An AE is any untoward medical occurrence in a clinical investigation subject, temporally associated with the use of a medicinal product, whether or not considered related to the medicinal product. An AE can therefore be any unfavourable and unintended sign (including an abnormal laboratory finding), symptom, or disease (new or exacerbated) temporally associated with the use of a medicinal product. For marketed medicinal products, this also includes failure to produce expected benefits (i.e. lack of efficacy), abuse or misuse.

Examples of an AE include:

- Significant or unexpected worsening or exacerbation of the condition/indication under study.
- Exacerbation of a chronic or intermittent pre-existing condition including either an increase in frequency and/or intensity of the condition.
- New conditions detected or diagnosed after investigational vaccines administration even though they may have been present prior to the start of the study.
- Signs, symptoms, or the clinical sequelae of a suspected interaction.
- Signs, symptoms, or the clinical sequelae of a suspected overdose of either investigational vaccines/ antimalarial medications or a concurrent medication (overdose per se should not be reported as an AE/SAE).
- Signs, symptoms temporally associated with vaccines/antimalarial drug administration.

- Pre- or post-treatment events that occur as a result of protocol-mandated procedures (i.e. invasive procedures, modification of subject's previous therapeutic regimen).

Examples of an AE DO NOT include:

- Medical or surgical procedures (e.g. endoscopy, appendectomy); the condition that leads to the procedure is an AE/SAE.
- Situations where an untoward medical occurrence did not occur (e.g. social and/or convenience admission to a hospital, admission for routine examination).
- Anticipated day-to-day fluctuations of pre-existing disease(s) or condition(s) present or detected at the start of the study that do not worsen.
- Pre-existing conditions or signs and/or symptoms present in a subject prior to the first study vaccination. These events will be recorded in the medical history section of the CRF.

1.2.2 Definition of a serious adverse event

A SAE is any untoward medical occurrence that:

- Results in death,
- Is life-threatening,

Note: The term 'life-threatening' in the definition of 'serious' refers to an event in which the subject was at risk of death at the time of the event. It does not refer to an event, which hypothetically might have caused death, had it been more severe.

- Requires hospitalization or prolongation of existing hospitalization,

Note: In general, hospitalization signifies that the subject has been admitted at the hospital or emergency ward for observation and/or treatment that would not have been appropriate in the physician's office or in an out-patient setting. Complications that occur during hospitalization are also considered AEs. If a complication prolongs hospitalization or fulfils any other serious criteria, the event will also be considered serious. When in doubt as to whether 'hospitalization' occurred or was necessary, the AE should be considered serious.

- Hospitalization for elective treatment of a pre-existing condition (known or diagnosed prior to informed consent signature) that did not worsen from baseline is NOT considered an AE.

- Results in disability/incapacity

Note: The term disability means a substantial disruption of a person's ability to conduct normal life functions. This definition is not intended to include experiences of relatively minor medical significance such as uncomplicated headache, nausea, vomiting, diarrhoea, influenza like illness, and accidental trauma (e.g. sprained ankle) which may interfere or prevent everyday life functions but do not constitute a substantial disruption.

- Is a congenital anomaly/birth defect in the offspring of a study subject.

- Spontaneous pregnancy loss

Medical or scientific judgment should be exercised in deciding whether reporting is appropriate in other situations, such as important medical events that may not be immediately life-threatening or result in death or hospitalization but may jeopardize the subject or may require medical or surgical intervention to prevent one of the other outcomes listed in the above definition. These should also be considered serious. Examples of such events are invasive or malignant cancers, intensive treatment in an emergency room or at home for allergic bronchospasm, blood dyscrasias or convulsions that do not result in hospitalization.

1.2.3 Solicited adverse events

Occurrence of solicited AE from the date of each vaccination to 7 days of each vaccination will be recorded.

Solicited local (injection-site) adverse events - The following local (injection-site) AEs will be solicited:

All age groups
Pain at injection site<br>Redness at injection site<br>Swelling at injection site<br>Bruising at injection site

Systemic solicited adverse events - The following general AEs will be solicited:

Adult
Fatigue<br>Fever<br>Diarrhoea<br>Nausea<br>Vomiting<br>Headache<br>Myalgia<br>Arthralgia<br>Chills

Note: Temperature will be recorded in the evening. Should additional temperature measurements be performed at other times of day, the highest temperature will be recorded in the CRF.

1.3 Clinical laboratory parameters and other abnormal assessments qualifying as adverse events or serious adverse events

In absence of diagnosis, abnormal laboratory findings (e.g. clinical chemistry, haematology, and urinalysis) or other abnormal assessments that are judged by the investigator to be clinically significant will be recorded as AE or SAE, if they meet the definition of an AE or SAE. Clinically

significant abnormal laboratory findings or other abnormal assessments that are present at baseline and significantly worsen following the start of the study will also be reported as AEs or SAEs. The investigator will exercise his or her medical and scientific judgment in deciding whether an abnormal laboratory finding or other abnormal assessment is clinically significant.

1.3.1 Adverse events of specific interest

AEs of specific interest for safety monitoring include meningitis and potential Immune-Mediated Diseases pIMDs.

- Potential immune-mediated diseases (pIMD)

pIMDs are a subset of AEs that include autoimmune diseases and other inflammatory and/or neurologic disorders of interest which may or may not have an autoimmune aetiology. AEs that need to be recorded and reported as pIMDs include those listed below. However, the investigator will exercise his/her medical and scientific judgment in deciding whether other diseases have an autoimmune origin (i.e. pathophysiology involving systemic or organ-specific pathogenic autoantibodies) and should also be recorded as a pIMD.

When there is enough evidence to make any of the above diagnoses, the AE must be reported as a pIMD. Symptoms, signs or conditions which might (or might not) represent the above diagnoses, should be recorded and reported as AEs but not as pIMDs until the final or definitive diagnosis has been determined, and alternative diagnoses have been eliminated or shown to be less likely.

Events or outcomes not qualifying as adverse events or serious adverse events

1.3.2 Pregnancy

Female volunteers who are pregnant or intend to become pregnant will not be enrolled. Female subjects who become unexpectedly pregnant during of vaccination period (M0 to M2) will not receive additional doses of study vaccines but may continue other study procedures at the discretion of the investigator. While pregnancy itself is not considered an AE or SAE, any adverse pregnancy outcome or complication or elective termination of a pregnancy for medical reasons will be recorded and reported as an AE or a SAE.

The following should always be considered as SAE.
Spontaneous pregnancy loss, including:

- spontaneous abortion, (spontaneous pregnancy loss before/at 22 weeks of gestation)
- ectopic and molar pregnancy
- stillbirth (intrauterine death of foetus after 22 weeks of gestation).
- Any early neonatal death (i.e. death of a live born infant occurring within the first 7 days of life).
- Any congenital anomaly or birth defect identified in the offspring of a study subject (either during pregnancy, at birth or later) regardless of whether the foetus is delivered dead or alive.

This includes anomalies identified by prenatal ultrasound, amniocentesis or examination of the products of conception after elective or spontaneous abortion.

Furthermore, any SAE occurring as a result of a post-study pregnancy AND considered by the investigator to be reasonably related to the investigational vaccines will be reported. While the investigator is not obligated to actively seek this information from former study participants, he/she may learn of a pregnancy through spontaneous reporting.

1.4 Detecting and recording adverse events and serious adverse events

1.4.1 Recording adverse events, serious adverse events and pregnancies

All AEs must be recorded into the appropriate section of the CRF, irrespective of intensity or whether or not they are considered vaccination-related.

1.4.2 Post-Study adverse events and serious adverse events

Investigators are not obliged to actively seek AEs or SAEs in former study participants. However, if the investigator learns of any SAE at any time after a subject has been discharged from the study, and he/she considers the event reasonably related to the investigational vaccines, the investigator will promptly notify the Study Contact for Reporting SAEs.

1.4.3 Active questioning to detect adverse events and serious adverse events

As a consistent method of collecting AEs, the subject should be asked a non-leading question such as: 'Have you felt different in any way since receiving the vaccines or since the previous visit?' When an AE/SAE occurs, it is the responsibility of the investigator to review all documentation (e.g. hospital progress notes, laboratory and diagnostics reports) relative to the event. The investigator will attempt to establish a diagnosis pertaining to the event based on signs, symptoms, and/or other clinical information. In such cases, the diagnosis should be documented as the AE/SAE and not the individual signs/symptoms.

1.5 Assessment of Adverse Events and Serious Adverse Events

1.5.1 Assessment of intensity

The intensity of the following solicited AEs will be assessed as described:

**Adverse Event**	**Parameter**
Redness at injection site	Record greatest surface diameter in mm
Swelling at injection site	Record greatest surface diameter in mm
Bruising at injection site	Record greatest surface diameter in mm
Fever*	Record temperature in °C/°F
Other AEs	Record grading

Severity grading criteria for the self-assessment for AEs.

GRADE 0	none
GRADE 1	noticeable, but no impact on activities
GRADE 2	noticeable and alters, but does not prevent daily activities
GRADE 3	prevents normal daily activities, and/or requires visits with a medical professional
GRADE 4	precipitates ER visit or hospitalization (these are, of course, also SAEs; Serious Adverse Effects).

*Fever is defined as temperature ≥ 37.5°C / 99.5°F for oral, axillary or tympanic route, or ≥ 38.0°C / 100.4°F for rectal route. The preferred route for recording temperature in this study will be oral.

The investigator and designated study staff will assess non-solicited adverse events and will grade them according to Common Terminology Criteria for Adverse Events (CTCAE) Version 5.0.
In the rare case that an adverse event is not graded in the CTCAE, then that event should be graded as follows:

Grade 1: Mild AE
Grade 2: Moderate AE
Grade 3: Severe AE
Grade 4: Life-threatening or disabling AE
Grade 5: Death related to AE

1.5.2 Assessment of causality

The investigator will assess the relationship between investigational vaccines and study antimalarial medications and the occurrence of each AE/SAE. The investigator will use clinical judgment to determine the relationship. Alternative plausible causes, such as natural history of the underlying diseases, concomitant therapy, other risk factors, and the temporal relationship of the event to the investigational vaccines and study antimalarial medications will be considered and investigated. The investigator will also consult the IB and/or Summary of Product Characteristics to determine his/her assessment. There may be situations when a SAE has occurred and the investigator has minimal information. However, it is very important that the investigator always makes an assessment of causality. The investigator may change his/her opinion of causality in light of follow-up information and update the SAE information accordingly. The causality assessment is one of the criteria used when determining regulatory reporting requirements. In case of concomitant administration of vaccine and study antimalarial medication, it may not be possible to determine the causal relationship of general AEs to the individual vaccine or medication administered. All solicited local (injection site) reactions will be considered causally related to vaccination. Causality of all other AEs should be assessed by the investigator using the following question:

Is there a reasonable possibility that the AE may have been caused by the investigational vaccine or study antimalarial medications?

YES	:	There is a reasonable possibility that the vaccines or study antimalarial medications contributed to the AE.
NO	:	There is no reasonable possibility that the AE is causally related to the administration of the study vaccines or study antimalarial medications. There are other, more likely causes and administration of the study vaccines and study antimalarial medications is not suspected to have contributed to the AE.

If an event meets the criteria to be determined as 'serious', additional examinations/tests will be performed by the investigator in order to determine ALL possible contributing factors for each SAE.

Possible contributing factors include:

- Medical history.
- Other medication.
- Protocol required procedure.
- Other procedure not required by the protocol.
- Lack of efficacy of the vaccines, if applicable.
- Erroneous administration.
- Other cause (specify).

1.5.3 Assessment of outcomes

The investigator will assess the outcome of all AEs (including SAEs) recorded during the study as:

- Recovered/resolved.
- Recovering/resolving.
- Not recovered/not resolved.
- Recovered with sequelae/resolved with sequelae.
- Fatal (SAEs only).

1.6 Safety (Analysis)

Safety and reactogenicity of the investigational vaccine will be analysed for the following

- Occurrence of solicited local and general AEs within seven days (day of vaccination and six subsequent days) after each vaccination.
- Occurrence of unsolicited AEs from the date of the first vaccination to 28 days after the last vaccination, according to the Medical Dictionary for Regulatory Activities (MedDRA) classification.
- Occurrence of SAEs (all, fatal, related to investigational vaccine) within 28 days (day of vaccination and 28 subsequent days) after each vaccination, according to the MedDRA classification.

- Occurrence of SAEs (all, fatal, related to investigational vaccine) during the whole study period according to the MedDRA classification.
- Occurrence of AEs and SAEs leading to withdrawal from further vaccination from Dose 1 (Day 0) up to study conclusion (Day 180), according to the MedDRA classification, for each vaccinated subjects.
- Occurrence of pIMDs from Dose 1 (Day 0) up to study conclusion (Day 180), according to the MedDRA classification, for each vaccinated subjects.
- Occurrence of meningitis from Dose 1 (Day 0) up to study conclusion (Day 180), according to the MedDRA classification, for each vaccinated subjects.

1.6.1 Analysis of safety

The percentage of subjects with at least one local AEs with at least one general AE (solicited and unsolicited) and with any AE during the 7-day or total follow-up period up to 28 days after the last vaccine dose and overall will be tabulated with exact 95% CI. The percentage of doses followed by at least one local AE (solicited and unsolicited), by at least one general AE (solicited and unsolicited) and by any AE during the 7-day or the total follow up period up to 28 days after the last vaccine will be tabulated with exact 95% CI. The same computations will be done for Grade 3 AEs, for any AEs considered related to vaccination and for any Grade 3 AEs considered related to vaccination.

The percentage of subjects reporting each individual solicited local AE (any grade and Grade 3) and solicited general AE (any grade, Grade 3, any related, Grade 3 related, resulting in medically attended visit) during the 7-day follow-up period (Day 0-6) after each vaccine dose and overall will be tabulated for each group. Similarly, the percentage of doses followed by each individual solicited local and general AE will be tabulated, overall vaccination course, with exact 95% CI.

For fever, the number and percentage of subjects reporting fever by half degree (°C) cumulative increments during the first seven days (Day 0-6) after each vaccine dose and overall will be tabulated. Similar tabulations will be performed for any fever with a causal relationship to vaccination and Grade 3 (> 39.5°C) causally related fever.

The percentage of subjects reporting unsolicited AEs from the date of the first vaccine dose up to 28 days after the last vaccine dose and after the sporozoite challenge will be tabulated by group and by MedDRA preferred term with exact 95% CI. Similar tabulation will be done for Grade 3 unsolicited AEs, for any causally related unsolicited AEs and for Grade 3 causally related unsolicited AEs.

The percentage of subjects reporting SAEs and pregnancies will be described in detail.

The percentage of vaccinated subjects reporting AEs of specific interest (meningitis and pIMDs) will be described in detail.

Biochemistry (ALT, AST and creatinine) and haematological (haemoglobin, WBC and platelets) laboratory values will be presented according to toxicity grading scales and tabulated by group.

1.7 Holding rules and safety monitoring

No group holding rules will be pre-defined considering the evidence for a favourable safety profile of this candidate vaccine in Phase 2I infant evaluation.

Individual study participants who present with at least one of the following stopping rules will be withdrawn from further vaccine and/or drug administrations:

- Local reactions: upon investigator discretion.
- Systemic solicited adverse events: The subject develops a Grade 3 systemic solicited adverse event beginning within 2 days after vaccination/drug administration (day of vaccination and one subsequent day) and persisting at Grade 3 for >2 consecutive days.
- Unsolicited adverse events: The subject has any Grade 3 adverse event considered related to vaccination, persisting at Grade 3 for >2 consecutive days.
- The subject has a serious adverse event considered related to vaccination.
- The subject has an acute allergic reaction or anaphylactic shock following the administration of vaccine investigational product.
- Laboratory adverse events: The subject has any Grade 3 laboratory adverse event considered related to vaccination.

1.8 Safety Oversight

1.8.1 Medical Monitor

A Medical Monitor, representing the Sponsor, will be appointed for oversight of safety in this clinical study. The Medical Monitor will be responsible for safety assessments as outlined below. The monitor will review the study prior to initiation and will be available to advise the Investigators on study-related medical issues and to act as a representative for the welfare of the subjects. The monitor does not have direct involvement in the conduct of the study and does not have other interests with any collaborating pharmaceutical firms or their competitors. All serious adverse events and any AEs that fulfil the criteria for pausing or halting will be reported to the medical monitor within 24 hours of becoming aware of the event. The monitor is responsible for the review of the safety data and communication with the PI, and/or the Data Safety Monitoring Board (DSMB), as appropriate. The Medical Monitor may also provide recommendation for continuation, modification, or termination of the study, if there is urgent need.

1.8.2 Data and Safety Monitoring Board

The Data Safety Monitoring Board (DSMB) will review the study prior to initiation and review all SAEs at the end of the study. The Board may convene additional reviews if deemed necessary, on review of the safety data, as sent, periodically by the Medical Monitor. All SAEs will be reported

by the PI to the DSMB at the same time they are submitted to the Institutional review board (IRB). The PI will notify the DSMB at the time pausing, or halting criteria are met, and obtain a recommendation concerning continuation, modification, or termination of the study. The PI will submit the written DSMB summary reports with recommendations to the IRB(s).

2 ADMINISTRATIVE INFORMATION

2.1 Definition of Start and End of Trial

The start of the trial is defined as the date of the first vaccination of the first participant. The end of trial is the date of the last visit of the last participant, which will be a maximum of 6 months after recruitment and first vaccination of the last participant.

2.2. Ethics

Ethical approval will be sought prior to commencing the study through the relevant Research Ethics Committees. Indemnity for the trial will be provided by the University of Oxford. SAEs will be reported to the medical monitor, DSMB, ethics committees and the Sponsor. GCP training will be provided to all staff/investigators who have no valid training certificate prior to commencing the studies.

2.3. Declaration of Helsinki

The Investigators at each site will ensure that this study is conducted in accordance with the principles of the Declaration of Helsinki.

2.4. ICH Guidelines for Good Clinical Practice

The trial will adhere to the Research Governance policies of the University of Oxford and the ICH GCP.

2.5. Approvals

The protocol, informed consent form, participant information sheet, and other written participant information / materials will be submitted to appropriate Research Ethics Committees (RECs), and regulatory authorities for written approval. The Principal Investigator will submit and, where necessary, obtain approval from the above parties for all amendments to the original approved documents.

2.6. Participant Confidentiality

The trial staff will ensure that the participants' anonymity is maintained. The participants will be identified only by participants' ID number on the CRF and in any electronic databases. All documents will be stored securely and only accessible to trial staff and authorized personnel. Only the sponsor representative, investigators, the clinical monitor, authorized individuals from the, Jenner Institute, the ethical committee(s) and the regulatory authorities will have access to the

records. Data will be anonymized as soon as it is practical to do so. With subject's consent, subject's clinical data and results from blood analyses stored in our database may be shared with the Jenner Institute and other researchers in the future. However, the other researchers will not be given any information that could identify the subject.

2.7. Transparency in Research

Prior to the recruitment of the first participant, the trial will have been registered on a publicly accessible trial registry. Results will be uploaded to the registry within 12 months of the end of trial declaration by the responsible party. Where the trial has been registered on the public platform, the trial information will be kept up to date during the trial, and the responsible party will upload results to the public registry within 12 months of the end of the trial declaration.

2.8. Quality Control and Quality Assurance

The study will be conducted in accordance with the current approved protocol, ICH GCP, relevant regulations and standard operating procedures. Data will be evaluated for compliance with the protocol and accuracy in relation to source documents. Following written standard operating procedures, the independent monitors from Clinical Trials Support Group (CTSG), MORU will verify that the clinical trial is conducted and data are generated, documented and reported in compliance with the protocol.

2.9. Publication and Data Policy

All Investigators will be involved in reviewing drafts of the manuscripts, abstracts, press releases and any other publications arising from the study. Authorship will be determined in accordance with the International Committee of Medical Journal Editors (ICMJE) guidelines and other contributors will be acknowledged.

2.10. Sponsor and Insurance

Sponsor name: University of Oxford
Contact information: University Offices, Wellington Square, Oxford, OX1 2JD, United Kingdom

The study sponsor has a role in the design of the study; collection of data; management, analysis, and interpretation of data; writing of the report; and the decision to submit the report for publication.

The University of Oxford has a specialist insurance policy in place: - Newline Underwriting Management Ltd, at Lloyd's of London – which would operate in the event of any participant suffering harm as a result of their involvement in the research.

2.11. Financing

The funder, Medical Research Council, had a role in the design of the study. The funder will not have any role in its execution, analyses, and interpretation of data or decision to submit results.

APPENDIX C: LIST OF ABBREVIATIONS

ACT	Artemisinin combination therapy
AE	Adverse event
ALT	Alanine aminotransferase
anti-CS	Antibody to the *P. falciparum* circumsporozoite (CS) repeat domain
anti-HBs	Antibody to the hepatitis B surface antigen
Anti-HCV	Antibody to hepatitis C virus
AST	Aspartate aminotransferase
CI	Confidence interval
CREA	Creatinine
CRF	Case report form
CSP	Circumsporozoite protein of P. falciparum
CTU	Clinical Therapeutics Unit (CTU), Hospital for Tropical Diseases, Faculty of Tropical Medicine, Mahidol University
DHA-PIP	Dihydroartemisinin–piperaquine
DSMB	Data safety monitoring board
ECG	Electrocardiogram
ELISA	Enzyme linked immunosorbent assay
EPI	Expanded programme of immunisations
EPT	Endpoint titre
GCP	Good clinical practice
GMS	Greater Mekong Subregion
Hb	Haemoglobin
HBsAg	Hepatitis B surface antigen
HBV	Hepatitis B virus
HCV	Hepatitis C virus
Hep B	Hepatitis B
HIV	Human immunodeficiency virus
HLA	Human Leukocyte Antigen
IB	Investigator's brochure
ICF	Informed consent form
ICH	International Committee on Harmonization
ICMJE	International Committee of Medical Journal Editors
IEC	Independent ethics committee
IgG	Immunoglobulin G
IM	Intramuscular
IRB	Institutional review board
IU	International unit
kg	Kilogram

MedDRA	Medical Dictionary for Regulatory Activities
mg	Milligram
mL	Milliliter
ms	Millisecond
NANP<br>*P. falciparum*	Asn-Ala-Asn-Pro<br>*Plasmodium falciparum*
PFS	Pre-filled syringe
PI	Principal Investigator
PLT	Platelets
piMD	Potential immune-mediated disease
PQ	Primaquine
RTS,S	Particulate antigen, containing both RTS and HBs proteins
SAE	Serious adverse event
SLD-PQ	Single low dose Primaquine
SOP	Standard operating procedure
TME	Targeted malaria elimination
ULN	Upper limit of normal
WBC	White blood cells
WHO	World Health Organization

APPENDIX D: HISTORY OF AMENDMENTS

Amendment No.	Protocol Version No.	Date issued	Author(s) of changes	Details of Changes made

APPENDIX E: SUBMISSION PACKAGE INCLUDES

**No.**	**Submitted Documents**	**No. of copy**
1	Cover letter from Principal Investigator's Department or Unit	1
2	Receipt of Submission Fee	1
3	Research Proposal Submission Form for a study involving human subject enrollment WITH specimen collection (FTM ECF-033-01)	1
4	Full research protocol or main protocol	1
5	Informed Consent Form, and Participant Information Sheet	1
6	Appointment Card	1
7	Subject Diary Card	1
8	Flyer	1
9	Screening Form	1

10	Case Record Form	1
11	Material Transfer Agreement (if applicable)	1
12	Investigator's Brochure	1
